# mGluR5 in Pyramidal Neurons in the Hippocampus Mediates Chronic Stress‐Induced Memory Deficits

**DOI:** 10.1111/cns.70477

**Published:** 2025-06-12

**Authors:** Hong‐Cheng Lu, Zhuo‐Jun Du, Hao Chen, Ting Guo, Shu‐Cai Yang, Xin Li

**Affiliations:** ^1^ Pingshan District Peoples' Hospital of Shenzhen Shenzhen Guangdong People's Republic of China; ^2^ Pingshan Hospital Southern Medical University Shenzhen Guangdong People's Republic of China; ^3^ Key Laboratory of Mental Health of the Ministry of Education, Guangdong‐Hong Kong‐Macao Greater Bay Area Center for Brain Science and Brain‐Inspired Intelligence, Guangdong‐Hong Kong Joint Laboratory for Psychiatric Disorders, Guangdong Province Key Laboratory of Psychiatric Disorders, Guangdong Basic Research Center of Excellence for Integrated Traditional and Western Medicine for Qingzhi Diseases, Department of Neurobiology, School of Basic Medical Sciences Southern Medical University Guangzhou China; ^4^ State Key Laboratory of Organ Failure Research, Institute of Brain Diseases, Nanfang Hospital Southern Medical University Guangzhou China

**Keywords:** chronic stress, memory deficits, mGluR5, PACAP, synaptic inputs

## Abstract

**Background:**

Chronic stress causes variable profiles of physiological deficits, including mood disorders, sleep disorders, and memory deficits. However, the neural mechanisms and potential drug targets of chronic stress‐induced memory deficit remain elusive.

**Aims:**

This study aimed to explore the function and regulatory mechanisms of metabotropic glutamate receptor 5 (mGluR5) in chronic stress‐induced memory deficit and investigate the potential therapeutic target for stress‐related memory deficit.

**Methods:**

Behavioral tests were used to assess the effects of chronic stress on memory. Electrophysiological recordings were conducted to examine the synaptic inputs after chronic stress. RNA sequencing was employed to achieve transcriptional alterations in the hippocampus after stress or mGluR5 knockdown. Enrichment analysis was performed to identify the downstream effector of chronic stress‐induced memory deficits.

**Results:**

Chronic restraint stress (CRS) impairs hippocampal‐dependent memory and electrophysiological recordings reveal that chronic stress impairs synaptic inputs. Subsequently, we observe that the mGluR5 level declines after CRS, which is an important molecule for learning and memory. mGluR5 knockdown induces memory deficits and impairs synaptic inputs. Enhancement of mGluR5 activity by CDPPB could restore chronic stress‐induced memory deficits and rescue impaired synaptic inputs. Furthermore, we identify that pituitary adenylyl cyclase activating peptide (PACAP) is down‐regulated after CRS and mGluR5 knockdown. PACAP application could restore the impaired inhibitory synaptic inputs after CRS.

**Conclusions:**

These results illuminate that the mGluR5 mediates chronic stress‐induced memory deficits, which may provide promising strategies for treating stress‐related memory deficits.

## Graphical abstract

Diagrammatic representation of the role of mGluR5 in controlling memory impairments brought on by chronic stress. Chronic stress caused memory impairment, decreased GABAergic synaptic inputs, and decreased mGluR5 and PACAP levels in the hippocampus CA1. CDPPB restored memory damage by reversing the decline in GABAergic synaptic inputs caused by the stress. The reduction in GABAergic synaptic inputs brought on by chronic stress could also be reversed by PACAP administration.

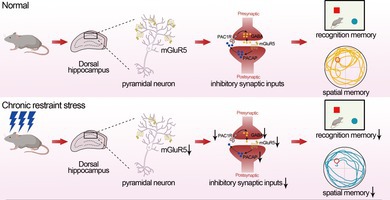

## 1 Introduction

Memory is among the most important abilities for higher organisms [1, 2, 3]. In humans and mice, chronic stress is a common risk factor for memory deficits, which could accelerate disease progression in many types of memory disorders [4, 5, 6, 7, 8, 9]. In recent years, studies have revealed the basic molecular and cellular mechanisms underlying memory formation and retrieval, providing new insights into the therapy of memory disorders [10, 11]. However, the neural mechanisms and potential drug targets for chronic stress‐induced memory deficits remain elusive.

The hippocampus is an area of the central nervous system known for regulating learning, memory, and emotion [12, 13, 14, 15, 16]. Evidence indicates that chronic stress could impair hippocampal function, including altered synaptic transmission, excitability, neurogenesis, neuronal morphology, and cell death [17, 18, 19, 20, 21]. Therefore, the hippocampus is also acknowledged as a crucial area that participates in the regulation of stress‐impaired learning and memory [17, 18, 19, 20, 21]. Currently, long‐term potentiation (LTP) is recognized as the major cellular mechanism for memory storage [16, 22]. However, accumulating evidence shows that neuronal excitability and inhibitory circuits are also important in memory storage [22, 23, 24]. Previous work suggests that inhibitory synaptic input, providing feedforward and feedback inhibition to pyramidal cells, is crucial for memory formation [16]. Together, multiple cellular mechanisms may participate in the process of memory formation.

mGluR5 (metabotropic glutamate receptor 5) is positively coupled to the PLC (phospholipase C) pathway [25, 26, 27]. mGluR5 activity could modulate vulnerability to chronic social stress [28]. mGluR5 plays a crucial role in regulating synaptic transmission, synaptic plasticity, learning, and memory [29, 30, 31, 32, 33, 34, 35]. Our previous work suggests that mGluR5 could regulate GABA release and anxiety‐like behaviors [21]. Previous studies show that mGluR5 was important for hippocampal plasticity and the acquisition of object‐place configurations [30]. mGluR5 KO mice have impaired LTP, learning, and memory in the behavioral tests [32]. Nevertheless, the function of mGluR5 in chronic restraint stress‐impaired memory remains largely unknown.

Pituitary adenylyl cyclase activating peptide (PACAP) is a neuropeptide known to be important for modulating synaptic transmission, synaptic plasticity, and memory‐related behaviors [36, 37, 38, 39, 40]. Generally, PACAP is released from the presynapse, while some studies indicated that PACAP existed in the postsynapse and might be released from the postsynapse [41]. Moreover, PACAP could potentiate GABA release via its actions at the presynaptic PAC1 receptor [42, 43]. PACAP application could enhance hippocampal synaptic plasticity and improve memory performance in Huntington's disease and Alzheimer's disease [36].

Stress effects on molecular, cellular, and behavioral alterations are different based on the frequency of restraint stress [33]. As reported, spatial memory was impaired after 10 days of restraint stress, but not less than 10 days [33]. Our previous work reveals that 5 days of restraint stress reduces mGluR5 level, impairs synaptic inputs in the hippocampal CA1 region, and induces anxiety‐like behavior, but not memory deficits [21]. However, in this work, we observed that 10 days of restraint stress reduced mGluR5 level, decreased PACAP level, impaired synaptic inputs in the hippocampal CA1 region, and induced hippocampal‐dependent memory deficits. From our perspective, both mGluR5 level and PACAP level decreases are necessary for the 10 days of restraint stress‐induced memory deficits, while mGluR5 level decrease, but normal PACAP level, might exist for the 5 days of restraint stress‐induced anxiety‐like behavior. Therefore, different from our previous work, this study suggested that mGluR5 could regulate 10 days stress‐induced memory deficits. This work may shed light on the treatment of memory‐related diseases.

## 2 Materials and Methods

### 2.1 Animal

All male mice (C57BL/6 background, aged 8 weeks) were kept in a temperature‐controlled environment (21°C–25°C) in conventional laboratory cages, three to four per cage, with a 12‐h standard light/dark cycle. In every experiment, only male mice were used. Every procedure was performed in compliance with the Animal Care Guidelines of the Chinese Council. Meanwhile, every procedure was approved by the Southern Medical University Animal Ethics Committee. The Jackson Lab provided the mGluR5 loxP mice (male, C57BL/6 background, aged 8 weeks). The mGluR5 KO mice (C57BL/6 background, aged 8 weeks) were achieved by crossing mGluR5 loxP mice with CMV‐Cre mice. From the Guangdong Medical Laboratory Animal Center (Guangzhou, China), 8‐week‐old C57BL/6 mice were acquired.

### 2.2 Chronic Restraint Stress

Every mouse was randomized to either the stress or control condition in its home cages. Every restriction took place between 10:00 and 12:00 in the morning. Each mouse was placed in a polypropylene conical tube with enough ventilation, and the tube top was fastened. This process was performed once a day for 10 days.

### 2.3 Drugs and Antibodies

We acquired drugs for electrophysiological recordings from Sigma or Tocris Bioscience. KCl (P5405, Sigma), Glucose (G7021, Sigma), NaH_2_PO_4_ (71505, Sigma), Sucrose (V900116, Sigma), MgSO_4_ (793612, Sigma), NaHCO_3_ (S5761, Sigma), CaCl_2_ (C5670, Sigma), NaCl (S5886, Sigma), Mg‐ATP (A9187, Sigma), Na‐GTP (51120, Sigma), CsCl (V900481, Sigma), MgCl_2_ (208337), QX314 (1014/100, Tocris). We bought PACAP (TP1878) and CDPPB (T22641) from Topscience. The final concentration was 0.05% or less when solutions were prepared using DMSO (D8418, Sigma). mGluR5 antibody (ab76316) was purchased from Abcam. β‐actin antibody (66009‐1‐Ig) was purchased from Proteintech. Rabbit anti‐Homer1 (160003), rabbit anti‐VGAT (131013), mouse anti‐Gephyrin (147011), and Guinea pig anti‐VGluT1 (135318) were bought from Synaptic Systems.

### 2.4 Spontaneous Alternation Y‐Maze Test

The mice were handled for 3 days before the beginning of behavioral tests. It has been confirmed that the Y‐maze test is a spatial working memory test. Three black arms measuring 35 × 5 × 10 cm made up the Y‐maze. After being positioned at the same end of one arm, each mouse was free to move throughout the maze. The arm entry is considered when the distance is less than 5 cm between mice and the end of the arm. Re‐entries to the same arm in the two successive arm entries are discounted. Both the order in which the mice entered and the number of entries were noted. In the three successive arm entries, when the mouse gets into a different arm of the labyrinth, this is known as spontaneous alternation.

### 2.5 Novel Object Recognition Test

The opaque apparatus measuring 25 × 25 × 25 cm (long × width × height) was utilized. The mice were acclimated to the device for 10 min on the first day (Day 1). The mice were exposed to a pair of identical objects for 10 min during the training session (Day 2). The testing trial was carried out 24 h later (Day 3), and a new object that differed in size, color, and shape was used in place of the old object. Exploration was measured using any inquisitive activity toward (head orientation, sniffing) or intentional contact with an object. Five minutes were allotted for the test. The percentage of exploration time devoted to the new object was determined to be the novel object recognition preference [preferred = exploration time for new objects/total exploration time]. 70% ethanol was used to clean the equipment in between sessions.

### 2.6 Morris Water Maze

This test was used to examine memory‐related behavior that is dependent on the hippocampus. A 1.2‐m‐diameter pool and a 10‐cm‐diameter platform make up the water maze. Three visual cues were hidden behind a white circular curtain that encircled the pool. One centimeter below the water lay the white escape platform. There were eight sections to the pool. The beginning locations were the remaining places along the pool's edge, with the exception of the four points next to and across from the platform. The mice received training for 4 days in a row and four trials per day to search for the hidden platform per trial. The mice were manually directed to the platform if they could not find it in 90 s. On the fifth day, the hidden platform was taken out and the mice were positioned across from it. They were then given points for how long they spent in the target quadrant and how many times they crossed the platform in less than 90 s. The temperature of the maze was maintained at 22°C.

### 2.7 RNA Sequencing

The Novogene Bioinformatics Institute in Beijing, China, carried out the RNA‐seq. Bilateral hippocampus CA1 sections were obtained fresh and flash‐frozen on dry ice after the brains were quickly removed. The RNA Nano 6000 was used to purify total RNA and evaluate its integrity. To acquire mRNA from total RNA, magnetic beads were used. In the First Strand Synthesis Reaction Buffer, divalent cations were used for fragmentation at high temperatures. M‐MuLV Reverse Transcriptase and a random hexamer primer were used to create first‐strand cDNA, and RNase H was used to break down the RNA. Then, using DNA Polymerase I and dNTP, second‐strand cDNA synthesis was carried out. Then, the Agilent Bioanalyzer was applied to evaluate the quality of the library and purify the PCR products. Using an Illumina Novaseq platform following cluster generation, the library preparations were sequenced.

### 2.8 Electrophysiological Recordings

Male adult mice were induced into anesthesia using ethyl ether. Their brains were promptly excised and placed into oxygenated modified, ice‐chilled artificial cerebrospinal fluid (ACSF) with the following composition: 2.5 mM potassium chloride, 10 mM glucose, 1 mM sodium phosphate monobasic, 220 mM sucrose, 2.5 mM magnesium sulfate, 26 mM sodium bicarbonate, and 1.3 mM calcium chloride. VT‐1200S vibratome (Leica) was used to prepare brain slices. Then the slices were placed into a holding chamber filled with standard ACSF, which included 2.0 mM calcium chloride, 2.5 mM potassium chloride, 10 mM glucose, 120 mM sodium chloride, 2.0 mM magnesium sulfate, 26 mM sodium bicarbonate, and 1.2 mM sodium phosphate monobasic. 95% O_2_ and 5% CO_2_ were used to continually bubble all solutions. Brain slices were placed in a recording chamber that was kept between 32°C and 34°C and perfused with ACSF at a rate of 2 mL per minute during the tests. Hippocampal pyramidal neurons were used for whole‐cell patch‐clamp recordings. Micropipettes with resistances ranging from 3 to 6 MΩ were used. A solution (pH 7.40, 285 mOsm) including 10 mM phosphocreatine, 4 mM Mg‐ATP, 0.3 mM Na‐GTP, 10 mM Hepes, 5 mM CsCl, 0.2 mM EGTA, 1 mM MgCl_2_, 125 mM Cs‐methanesulfonate, and 5 mM QX314 was added into glass pipettes for sEPSC and mEPSC recordings in the presence of the GABAAR antagonist BMI (20 μM). For sEPSC and mEPSC recordings, the experiments were in voltage‐clamp condition, and the holding potential was −70 mV. A solution comprising 0.5 mM CaCl_2_, 110 mM Cs_2_SO_4_, 5 mM EGTA, 2 mM MgCl_2_, 5 mM HEPES, 5 mM ATP‐Mg, 5 mM TEA, pH 7.35, 285 mOsm, and holding potentials of 0 mV was used for sIPSC and mIPSC recordings in the presence of AP5 (50 μM) and CNQX (20 μM). The bath solution was supplemented with 1 μM TTX for mEPSC and mIPSC recordings. For CDPPB and PACAP application (acute drug perfusion) in slices, the incubation time was 10 min. In short, we performed the first recording before CDPPB or PACAP application. Then, the drugs were incubated for 10 min, and we conducted the second recording. For recordings, the holding current was kept less than 80 pA, and the change of holding current was less than 10% during the recordings. The access resistance was kept less than 25 MΩ. For analysis, the events whose amplitudes were less than 10 pA were not taken into account.

### 2.9 Western Blot

Using ice‐cold RIPA lysis buffer (Thermo Fisher Scientific, USA) with 1x protease inhibitors (Thermo Fisher Scientific), the tissue was extracted and lysed. Using SDS‐PAGE, the total protein was then separated and transferred onto a PVDF membrane. After blocking the membrane for 1 h at room temperature with 5% defatted milk powder, the main antibody was used for immunoblotting at 4°C overnight. It was then incubated for 1 h at room temperature with a secondary antibody (1:5000, Abbkine) conjugated with HRP. To visualize the blots, a Pierce super‐enhanced chemiluminescence (ECL) system was used. Image Lab software was used to determine the gray density of the bands in order to assess the levels of protein expression. Internal controls were used to standardize the results.

### 2.10 qPCR

The PrimeScript RT reagent kit (Takara, RR037A) was used for reverse transcription, and SYBR Premix Ex Taq (Takara, RR420A) was used for RT‐qPCR studies. Every step was completed in compliance with the manufacturer's guidelines. The mGluR5 primer set was created using Primer‐BLAST (NCBI). For real‐time RT‐PCR, the primer sequences listed below were employed. Forward GAPDH: 5′‐CAA TGT GTC CGT CGT GGA TCT‐3′; reverse GAPDH: 5′‐GTC CTC AGT GTA GCC CAA GAT G‐3′. To examine the qPCR results, the ∆∆Ct method was used. GAPDH was used as a reference gene.

### 2.11 Stereotaxic Microinjection

After receiving 1% pentobarbital sodium anesthesia, the mice were then put into a stereotaxic frame. A syringe pump was used to gradually deliver a Hamilton needle filled with virus into the dorsal hippocampus CA1 (AP, −2 mm; ML, −1.6 or +1.6 mm; DV, −1.6 mm). At a rate of 0.05 μL/min, bilateral injections of 0.2 μL of virus were made. The needle was left in for 10 min before being taken up to give the AAV time to diffuse. Following their recovery on an electric blanket and removal from the stereotaxic device, the animals were returned to their individual cages. Recovery from surgery typically took at least 3 weeks. Behavior tests and electrophysiological recordings were conducted 21 days following viral transduction. BrainVTA (Wuhan, China) created the CamKII Cre virus (serotypes: 2/9; titer: 3.1 × 1013; promoter: CamKIIa).

### 2.12 PACAP Level Measurement

To measure the level of PACAP, bilateral hippocampal CA1 regions were acquired. Using RIPA lysis buffer, the tissue was lysed. PACAP levels were measured using an ELISA kit (Jianglaibio, JL17323).

### 2.13 Slice for Confocal Microscopy

The mice have been anesthetized with 1% pentobarbital sodium and given a transcardial infusion of paraformaldehyde (4%) in PBS (0.1 M, pH = 7.4) 3 weeks following viral transduction. After being post‐fixed for a whole night, the brains were rinsed with water for an hour and preserved in 30% sucrose (wt/vol). On a cryostat, each brain was divided into coronal pieces measuring 40 μm. The Nikon A1 confocal microscope was used to capture a number of fluorescent images. Slices were blocked in 5% BSA (vol/vol) after being washed in 0.1 M PBS for immunofluorescence labeling. Sections were then incubated in the primary antibody in PBS for an entire night at 4°C. On Day 2, the Alexa secondary antibody was incubated for 2 h. Using the A1 Nikon confocal microscope, a number of fluorescent pictures were captured for additional examination with NIS‐Elements (Nikon) software. For synaptic staining, the synapse number was quantified by co‐localization of pre and postsynaptic proteins at synaptic junctions due to their close proximity. Using an A1 Nikon confocal microscope, high magnification images with an objective of 60× were captured.

### 2.14 Intracerebral Infusions

One percent pentobarbital sodium was used to put the mice to be anesthetized. Guide cannulas made of stainless steel (RWD; 62004; 2 mm in length) were inserted into the CA1 area of the dorsal hippocampus. Glass ionomer cement was used to hold the guiding cannulas in place. Glass ionomer cement was used to seal and hide the incisions. After being revived on an electric blanket, the mice were returned to their original cages. Behavior tests were conducted 7 days following surgery, following the treatment of ACSF or CDPPB. At a rate of 0.1 μL/min, bilateral injections of 0.3 μL of CDPPB were made.

### 2.15 Statistical Analyses

Version 7.0 of GraphPad Prism was used to conduct the statistical analysis. For every experiment, *p* < 0.05 was set as the threshold for statistical significance. The distribution of the data was assessed for normality using the Shapiro–Wilk test. When the data was normally distributed, the Student's *t*‐test was used to analyze data from two groups. Data from three or more groups were analyzed using a one‐way ANOVA. When examining more than two parameters, a two‐way ANOVA was employed. When the data was not normally distributed, a nonparametric test was utilized. The Mann–Whitney test was used to analyze data from two groups. The escape latency in the Morris water maze test was assessed using a repeated measure two‐way ANOVA with “day” as the within‐subject factor and “group” as the between‐subject factor, with repeated measures on days. In electrophysiological recordings, the synaptic inputs before or after drug application were assessed using a repeated measure two‐way ANOVA with “time” as the within‐subject factor and “group” as the between‐subject factor, with repeated measures on time. The mean ± SEM was displayed for the data.

## 3 Results

### 3.1 Chronic Stress Impaired Hippocampal‐Dependent Memory and Synaptic Inputs

**FIGURE 1:**
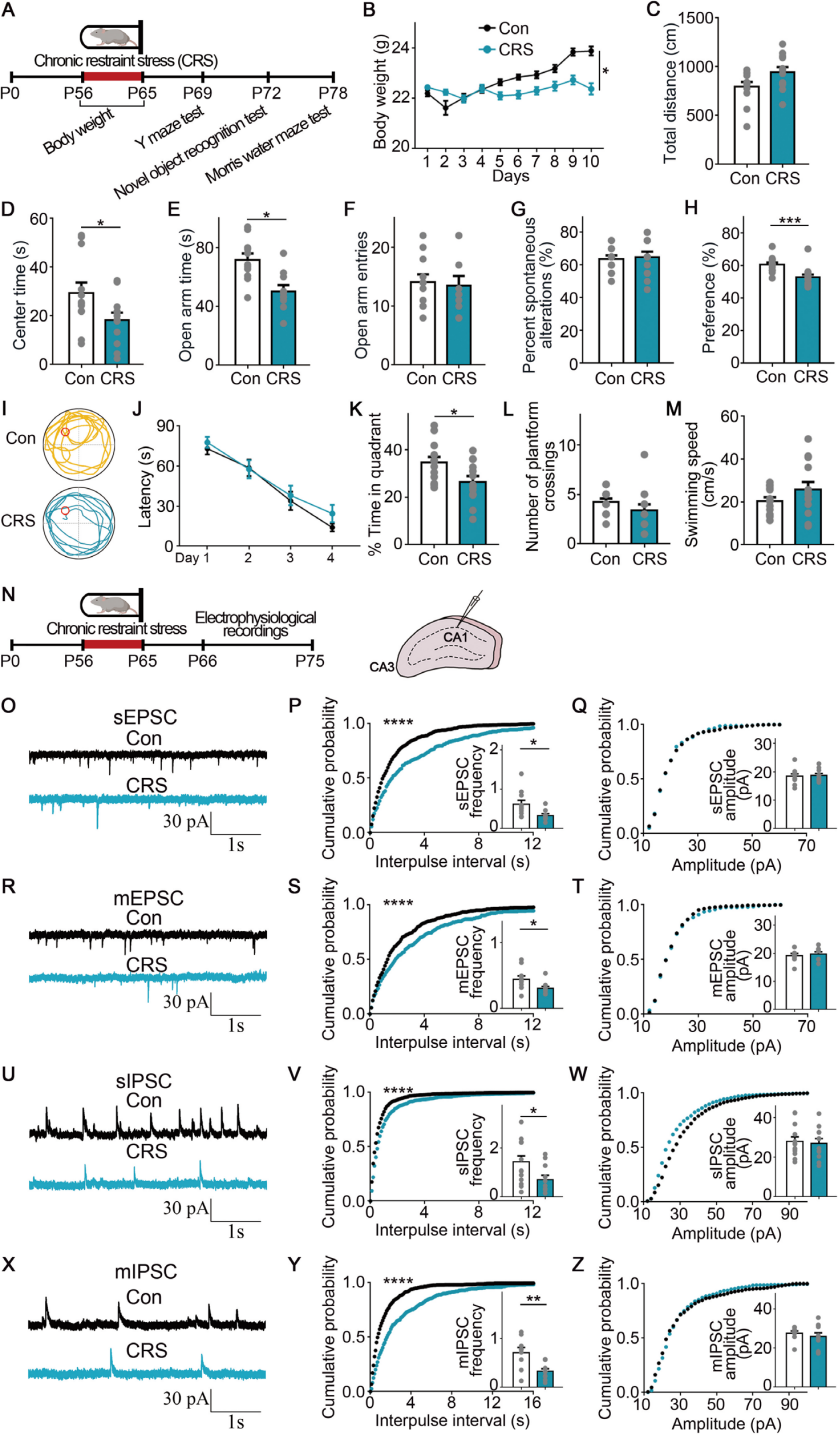
Chronic restraint stress impaired hippocampal‐dependent memory and synaptic inputs. (A) Diagram of behavioral assessments following chronic stress. (B) The mice were weighed daily. *n* = 13 for each group. (C, D) In the open field test, the mice subjected to chronic stress showed reduced center time. *n* = 12 for each group. (E, F) In the elevated plus maze test, the mice subjected to chronic stress showed reduced open arm time. *n* = 10–11 mice. (G) In the Y‐maze test, the mice subjected to chronic stress did not significantly differ from the control mice. *n* = 13 for each group. (H) In the novel object recognition (NOR) test, the mice exposed to chronic stress exhibited a decreased preference for the novel object. *n* = 13 for each group. (I) In the Morris water maze (MWM) test, the representative swimming traces followed chronic stress during the probe test. (J) In the training phase of the MWM test, there was no significant difference between the groups in the latency to find the hidden platform. *n* = 13 for each group. (K) In the probe test of the MWM test, the mice subjected to chronic stress exhibited impaired memory. (L, M) In the platform crossings and swimming speed of the Morris water maze, the mice subjected to chronic stress did not significantly differ from the control mice. (N) Diagram of electrical recordings in CA1 pyramidal neurons in the hippocampus. (O–Q) In sEPSC recordings, quantitative analysis of frequency and amplitude after chronic stress. 4 mice, *n* = 10–11 cells. (R–T) In mEPSC recordings, quantitative analysis of frequency and amplitude after chronic stress. 4 mice, *n* = 9–10 cells. (U–W) In sIPSC recordings, quantitative analysis of frequency and amplitude after chronic stress. 4 mice, *n* = 11–13 cells. (X–Z) In mIPSC recordings, quantitative analysis of frequency and amplitude after chronic stress. 4 mice, *n* = 9–11 cells. Error bars, mean ± SEM. *p < 0.05, **p < 0.01, ***p < 0.001, ****p < 0.0001. Scale bar: 1 s, 30 pA.

In our work, to determine if chronic stress could impair learning and memory in mice, we adopted chronic restraint stress (CRS) (Figure 1A) [21]. Mice (C57BL/6 background, aged 8 weeks) then underwent different behavioral tests (Figure 1A). Consistent with previous findings, we observed that mice exhibited decreased body weight after CRS (*F*_(1,24)_ = 6.087, *p* = 0.0211, *repeated measure two‐way ANOVA*; Figure 1B) [21]. Moreover, in the open field test, the CRS‐treated mice spent less time in the center area than the control group (*t*(22) = 2.091, *p* = 0.0483, *Student's t‐test*; Figure 1C,D). In the elevated plus maze test, the CRS‐treated mice showed less time in the open arm (*t*(19) = 3.509, *p* = 0.0023, *Student's t‐test*; Figure 1E,F). Together, these results indicated that the CRS‐treated mice exhibited anxiety‐like behaviors.

In the spontaneous alternation percentage of the Y‐maze test, we did not observe a significant difference between the groups, indicating that spatial working memory was not impaired after CRS (Figure 1G). However, in the novel object recognition (NOR) test, the CRS‐treated mice exhibited impaired object recognition, as evidenced by the decreased preference for the novel object when compared with control mice (*p* = 0.0003, Mann–Whitney test; Figure 1H). In the Morris water maze (MWM) test, the latency to search for the hidden platform did not exhibit a significant difference between the groups during the training phase (Figure 1J). However, during the probe test, the mice after stress spent less time in the target quadrant when compared with the control mice (*t*(24) = 2.459, *p* = 0.0215, *Student's t‐test*; Figure 1I,K). In addition, the CRS‐treated mice did not display deficits in swimming velocity or crosses over the absent platform during the Morris water maze, suggesting that impaired spatial memory was not due to swimming ability (Figure 1L,M). The memory deficits in the NOR test and MWM test might be associated with the function of the hippocampus [44, 45].

We performed electrophysiological recordings to determine the cellular mechanism of memory impairment following chronic stress (Figure 1N). We looked into whether there were any changes to the glutamatergic and GABAergic inputs in hippocampal CA1 pyramidal neurons after chronic stress. We observed that the average values of frequency both reduced in sEPSC and mEPSC recordings after chronic stress (*sEPSC*: *p* = 0.0127, *Mann–Whitney test*; *mEPSC*: *p* = 0.0262, *Mann–Whitney test*; Figure 1O–T). Similarly, in sIPSC and mIPSC recordings, while there was no change in the amplitude after chronic stress, the average values of frequency decreased following chronic stress (*sIPSC*: *p* = 0.0352, *Mann–Whitney test*; *mIPSC*: *t*(18) = 3.383, *p* = 0.0033, *Student's t‐test*; Figure 1U–Z).

Following chronic stress, the curves of cumulative frequency distribution in sEPSC and mEPSC showed a substantial drop when examined by the Kolmogorov–Smirnov (KS) test (*p* < 0.0001; Figure 1O–T). Meanwhile, following chronic stress, the curves of cumulative frequency distribution in sIPSC and mIPSC showed a substantial drop when examined by the KS test (*p* < 0.0001; Figure 1U–Z). Together, these findings suggested that synaptic inputs were impaired, which may be the cause of memory impairments following chronic stress. Given that the impaired synaptic inputs might be due to the reduced neurotransmitter release or the decreased synaptic density, we next determined whether synaptic density was altered after chronic restraint stress. Synapses were labeled with presynaptic and postsynaptic markers (excitatory: VGluT1 and Homer1, inhibitory: VGAT and Gephyrin). However, we did not observe an alteration in excitatory presynaptic density, excitatory postsynaptic density, inhibitory presynaptic density, or inhibitory postsynaptic density between the groups (Figure S1A–F). These results indicated that the impaired synaptic inputs after chronic restraint stress might be due to the reduced neurotransmitter release from presynapse, but not the structural alteration.

### 3.2 Chronic Stress Decreased mGluR5 Level and mGluR5 Knockdown Impaired Memory and Synaptic Inputs

**FIGURE 2:**
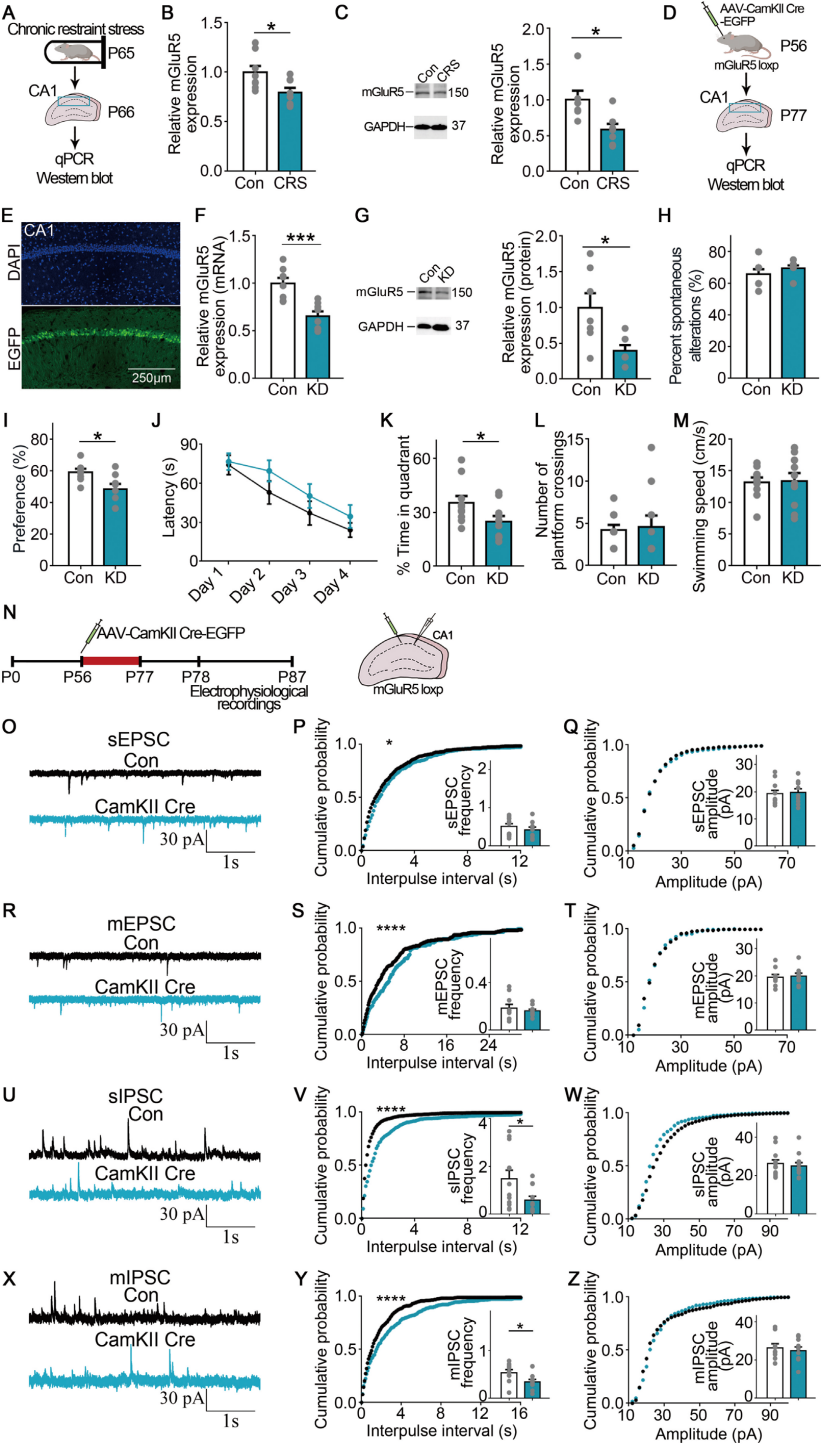
Chronic stress decreased mGluR5 level and mGluR5 knockdown impaired memory and synaptic inputs. (A) Diagram of qPCR and Western blot analysis following chronic stress. (B) The mice subjected to chronic stress exhibited decreased mGluR5 mRNA level in CA1. *n* = 8 for each group. (C) The mice subjected to chronic stress exhibited decreased mGluR5 protein level in CA1. There were 6–7 mice in each group. (D) Diagram of qPCR and Western blot analysis following virus injection. (E) The distribution of EGFP in the virus‐injected mice. Scale bar: 250 μm. (F) The mice exhibited decreased mGluR5 mRNA level in CA1 following virus injection. *n* = 8 for each group. (G) The mice exhibited decreased mGluR5 protein level in CA1 following virus injection. There were 6–7 mice in each group. (H) In the Y‐maze test, the mice did not significantly differ from the control mice following virus injection. *n* = 10 for each group. (I) In the NOR test, the mice exhibited a decreased preference for the novel object following virus injection. *n* = 8 for each group. (J) In the training phase of the MWM test, there was no significant difference between the groups in the latency to find the hidden platform following virus injection. *n* = 10 for each group. (K) In the probe test of the MWM test, the mice exhibited impaired memory following virus injection. (L, M) In the platform crossings and swimming speed of the Morris water maze, the mice did not significantly differ from the control mice following virus injection. (N) Diagram of electrical recordings in CA1 pyramidal neurons in the hippocampus following mGluR5 knockdown. (O–Q) In sEPSC recordings, quantitative analysis of frequency and amplitude after mGluR5 knockdown. 4 mice, *n* = 9–11 cells. (R–T) In mEPSC recordings, quantitative analysis of frequency and amplitude after mGluR5 knockdown. 4 mice, *n* = 9–10 cells. (U–W) In sIPSC recordings, quantitative analysis of frequency and amplitude after mGluR5 knockdown. 4 mice, *n* = 12 cells. (X–Z) In mIPSC recordings, quantitative analysis of frequency and amplitude after mGluR5 knockdown. 4 mice, *n* = 9–10 cells. Error bars, mean ± SEM. *p < 0.05, ***p < 0.001, ****p < 0.0001. Scale bar: 1 s, 30 pA.

Previous studies have established a notable causal link between mGluR5 and cognitive function, in which mGluR5 could regulate synaptic transmission, synaptic plasticity, learning, and memory [29, 30, 31, 32, 33, 34, 35]. Also, our previous work suggests that mGluR5 is involved in chronic stress‐induced anxiety‐like behaviors [21]. Therefore, we hypothesized that mGluR5 might mediate chronic stress‐induced memory deficits. To validate this hypothesis, we examined the mGluR5 level in the hippocampal CA1 region using quantitative PCR (Figure 2A). Remarkably, the mRNA level of mGluR5 in the CA1 region declined after chronic stress (*t*(14) = 2.566, *p* = 0.0224, *Student's t‐test*; Figure 2B). Meanwhile, compared with the control mice, the protein level of mGluR5 was reduced (*t*(11) = 2.784, *p* = 0.0178, *Student's t‐test*; Figure 2C).

We next investigated the potential loss‐of‐function effect of mGluR5 through the use of mGluR5 knockout (KO) mice and behavioral tests. We observed that the protein level of mGluR5 was undetectable after mGluR5 knockout (*p* = 0.0012, *Mann–Whitney test*; Figure S2A). In the Y‐maze test, mGluR5 KO mice did not show a significant difference with control mice in spontaneous alternation percentage, suggesting that spatial working memory was not altered by mGluR5 deletion (Figure S2B). However, in the novel object recognition test, mGluR5 KO mice exhibited impaired object recognition memory, as evidenced by the decreased preference for the novel object (*t*(18) = 2.637, *p* = 0.0167, *Student's t‐test*; Figure S2C). In the MWM test, the mGluR5 KO mice exhibited increased latency in finding the hidden platform when compared with control mice during the training phase (*F*_(1,18)_ = 4.853, *p* = 0.0409, *repeated measure two‐way ANOVA*; Figure S2D). Compared to the control mice, the mGluR5 KO mice spent less time in the target quadrant during the probe test (*p* = 0.0052, *Mann–Whitney test*; Figure S2E). A similar swimming velocity and crosses over the absent platform as that of the control mice were found in the mGluR5 KO mice (Figure S2F,G). Together, consistent with previous findings, these results indicated that global mGluR5 knockout impaired learning and memory.

To further determine if mGluR5 could regulate memory, using mGluR5 loxP/loxP mice, AAV‐CamKII‐Cre‐EGFP was developed and microinjected into the hippocampal CA1 region (Figure 2D). Three weeks later, in pyramidal neurons of the hippocampal CA1 region, EGFP was observed to be expressed (Figure 2E). Meanwhile, the mRNA and protein levels of mGluR5 both declined, demonstrating that mGluR5 was selectively knocked down in hippocampal CA1 pyramidal neurons (*mRNA*: t(14) = 4.535, *p* = 0.0005, *Student's t‐test*; *Protein*: *t*(11) = 2.647, *p* = 0.0227, *Student's t‐test*; Figure 2F,G). To investigate if mGluR5 knockdown impaired memory, we conducted behavioral tests. In the Y‐maze test, the spontaneous alternation percentage of the mGluR5 knockdown mice did not show a significant difference from the control mice, which indicated that spatial working memory was not impaired after mGluR5 knockdown (Figure 2H). In the NOR test, the mGluR5 knockdown mice showed reduced preference for the novel object when compared with control mice, indicating that object recognition memory was impaired after mGluR5 knockdown (*t*(14) = 2.713, *p* = 0.0168, *Student's t‐test*; Figure 2I). In the MWM test, the latency to search for the hidden platform showed no change between the groups during the training phase (Figure 2J). Compared to the control mice, the mice spent less time in the target quadrant after mGluR5 knockdown during the probe test (*t*(18) = 2.119, *p* = 0.0483, *Student's t‐test*; Figure 2K). The mice showed similar swimming velocity and crosses over the absent platform as that of the control mice (Figure 2L,M). In summary, these data indicated that mGluR5 knockdown impaired memory.

We performed electrophysiological recordings to determine the cellular mechanism of memory impairment following mGluR5 knockdown (Figure 2N). In sEPSCs and mEPSCs recordings, we did not observe significant differences in the frequency/amplitude between the groups (Figure 2O–T). However, in sIPSCs and mIPSCs recordings, while there was no change in the amplitude across the groups, the average values of frequency decreased following mGluR5 knockdown (*sIPSC*: *p* = 0.0387, *Mann–Whitney test*; *mIPSC*: *t*(17) = 2.272, *p* = 0.0364, *Student's t‐test*; Figure 2U–Z). Following mGluR5 knockdown, the curves of cumulative frequency distribution in sEPSC and mEPSC showed a substantial drop when examined by the KS test (*sEPSC*: *p* < 0.05; *mEPSC*: *p* < 0.0001; Figure 2O–T). Meanwhile, following mGluR5 knockdown, the curves of cumulative frequency distribution in sIPSC and mIPSC showed a substantial drop when examined by the KS test (*p* < 0.0001; Figure 2U–Z). Considering that the reduced extent of excitatory synaptic input was smaller than inhibitory synaptic input (only the mean values of IPSC showed substantial drop, but the mean values of EPSC were unaltered), these data indicated that inhibitory synaptic input was mainly impaired after mGluR5 knockdown. Furthermore, we conducted mEPSCs and mIPSCs recordings in mGluR5 KO mice and found that the GABAergic input in hippocampal CA1 pyramidal neurons was also impaired, while the glutamatergic input was unaltered (*mIPSC*: *p* = 0.0276, *Mann–Whitney test*; Figure S2H–K). These results indicated that inhibitory synaptic input was impaired after mGluR5 knockout.

Next, we explored if the CRS‐induced memory impairment was dependent on the role of mGluR5. We achieved the effects of CRS in mGluR5 knockdown mice (Figure S3A). In the Y‐maze test, we did not observe a significant difference between the groups, indicating that spatial working memory was not impaired after CRS in mGluR5 knockdown mice (Figure S3B). In the novel object recognition test and Morris water maze test, we did not observe memory deficits after CRS in mGluR5 knockdown mice, indicating that memory deficits could not be further increased by chronic stress in mGluR5 knockdown mice (Figure S3C–G).

### 3.3 CDPPB Rescued Impaired Synaptic Inputs and Memory Deficits Following Chronic Stress

**FIGURE 3:**
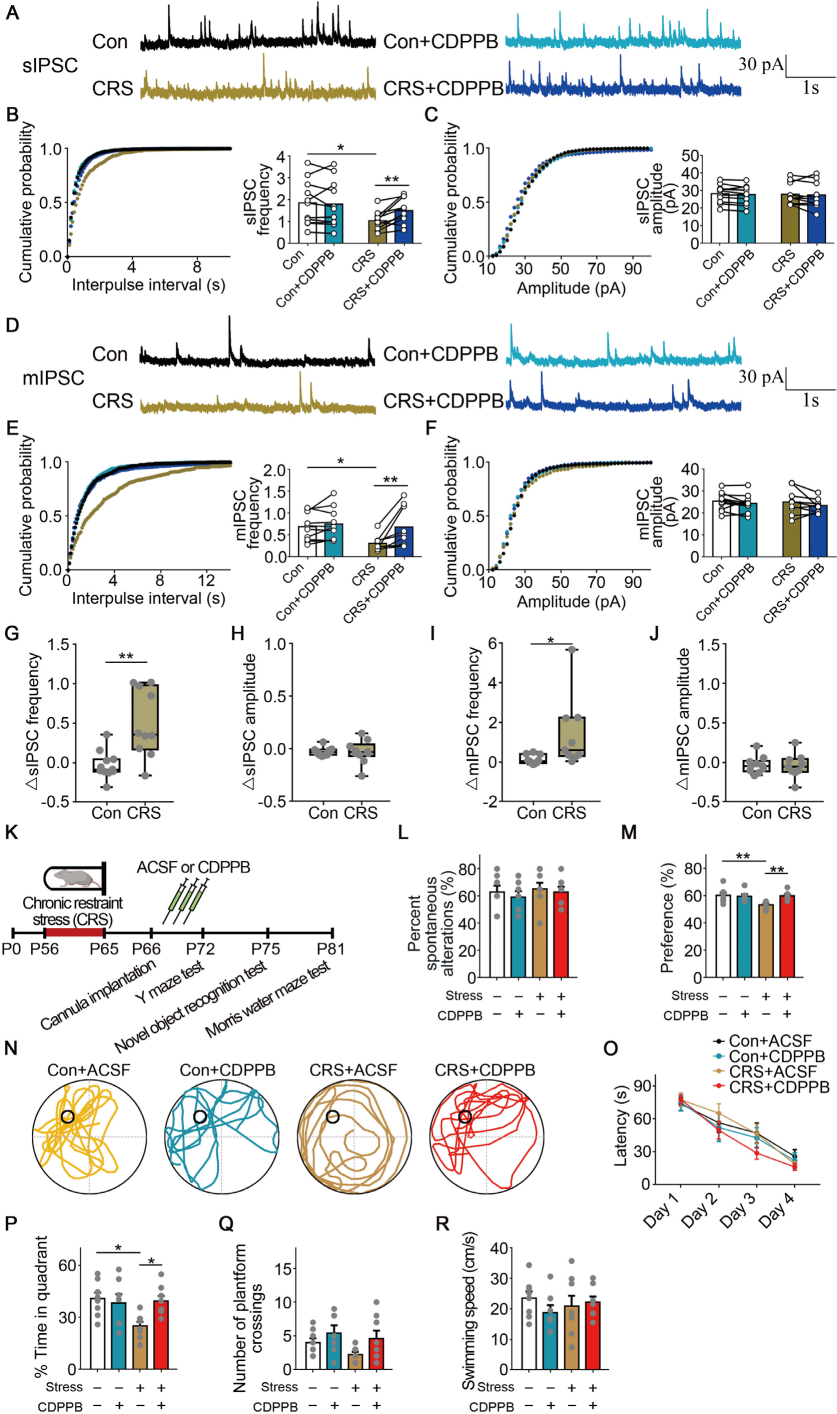
CDPPB rescued inhibitory synaptic inputs and memory deficits following chronic stress. (A–C) In sIPSC recordings, quantitative analysis of frequency and amplitude following CDPPB application. 5–6 mice, *n* = 10–11 cells. (D–F) In mIPSC recordings, quantitative analysis of frequency and amplitude following CDPPB application. 4–6 mice, *n* = 9–10 cells. (G, H) In sIPSC recordings, quantitative analysis of Δfrequency and Δamplitude following CDPPB application. 5–6 mice, *n* = 10–11 cells. (I, J) In mIPSC recordings, quantitative analysis of Δfrequency and Δamplitude following CDPPB application. 4–6 mice, *n* = 9–10 cells. (K) Diagram of behavioral assessments following chronic stress and CDPPB infusion. (L) In the Y‐maze test, the mice infused with CDPPB following chronic stress did not significantly differ from the control mice. There were 8, 7, 8, 8 mice, separately. (M) In the NOR test, the mice infused with CDPPB following chronic stress exhibited a restored preference for the new object. There were 8, 7, 8, 8 mice, separately. (N) In the MWM test, the representative swimming traces following CDPPB infusion during the probe test. (O–R) In the MWM test, the mice infused with CDPPB following chronic stress restored impaired memory. There were 8, 7, 8, 8 mice, separately. Error bars, mean ± SEM. *p < 0.05, **p < 0.01.

We next investigated if the increase of mGluR5 activity could restore synaptic input deficits following chronic stress. As reported, as a positive allosteric modulator for mGluR5, CDPPB may increase mGluR5 activity [46, 47, 48]. We explored if CDPPB might rescue impaired GABAergic synaptic inputs following chronic stress, given that the level of mGluR5 was reduced. Following chronic stress, we recorded GABAergic synaptic inputs in pyramidal neurons of hippocampus CA1. Our findings demonstrated that the frequency and amplitude of sIPSCs/mIPSCs in the control group were unaffected by 5 μM CDPPB (Figure 3A–F). While there was no change in the amplitude, 5 μM CDPPB raised the average values of frequency in the chronic stress group (*sIPSC*: *Con* vs. *CRS*: *p* = 0.0327. *CRS* vs. *CRS*(*CDPPB*): *p* = 0.0014. *Con* vs. *CRS*(*CDPPB*): *p* = 0.3461; *mIPSC*: *Con* vs. *CRS*: *p* = 0.0228. *CRS* vs. *CRS* (*CDPPB*): *p* = 0.0010. *Con* vs. *CRS*(*CDPPB*): *p* = 0.9616; *repeated measure two‐way ANOVA, Fisher's LSD test for multiple comparisons corrections*; Figure 3A–F). Following CDPPB treatment, the curves of cumulative frequency distribution in sIPSC and mIPSC showed a rescue when examined by the KS test in the chronic stress group (Figure 3A–F). Additionally, after applying CDPPB, we found that the chronic stress group had a higher sIPSC frequency (*p* = 0.0037, *Mann–Whitney test*), although there was no significant difference in the sIPSC amplitudes (Figure 3G,H). Similarly, following the CDPPB administration, we found that the chronic stress group had a higher mIPSC frequency (*p* = 0.0101, *Mann–Whitney test*), although there was no significant difference in the mIPSC amplitudes (Figure 3I,J). All of these findings suggested that following chronic stress, CDPPB could rescue the impaired GABAergic synaptic inputs.

Previous studies have demonstrated that, compared to vehicle‐treated controls, CDPPB significantly enhances memory and could improve search strategy in the Barnes maze [49]. Moreover, when applied to the mouse models of Alzheimer's disease, CDPPB produces neuroprotective effects [50]. CDPPB could also enhance adaptive learning and recognition memory [51, 52]. Therefore, we examined whether CDPPB could restore memory deficits after chronic stress. 5 μM CDPPB was infused into the CA1 region, and we conducted behavioral tests (Figure 3K).

In the Y‐maze test, the spontaneous alternation percentage did not significantly differ across the groups, suggesting that CDPPB infusion had no effect on spatial working memory (Figure 3L). In the novel object recognition test, the reduced preference for the novel object was rescued by CDPPB application, indicating that recognition memory was rescued (*Con* vs. *CRS*: *p* = 0.0094, *CRS* vs. *CRS + CDPPB*: *p* = 0.0058, *Kruskal–Wallis test, Dunn's test for multiple comparisons corrections*; Figure 3M). In the MWM test, the latency to search for the hidden platform showed no change between the groups during the training phase (Figure 3O). During the probe test, the infusion of CDPPB restored impaired memory after chronic stress (*F*_(3,27)_ = 4.231, *Con* vs. *CRS*: *p* = 0.0198, *CRS* vs. *CRS + CDPPB*: *p* = 0.0360, *One‐way ANOVA*, *Tukey test*; Figure 3N,P). Meanwhile, no significant difference was found in the swimming velocity or crosses over the absent platform (Figure 3Q,R). Together, these data indicated that CDPPB could restore memory deficits following chronic stress.

### 3.4 PACAP Was Predicted to Be the Downstream Effector of Chronic Stress and mGluR5 Knockdown‐Induced Memory Deficits

**FIGURE 4:**
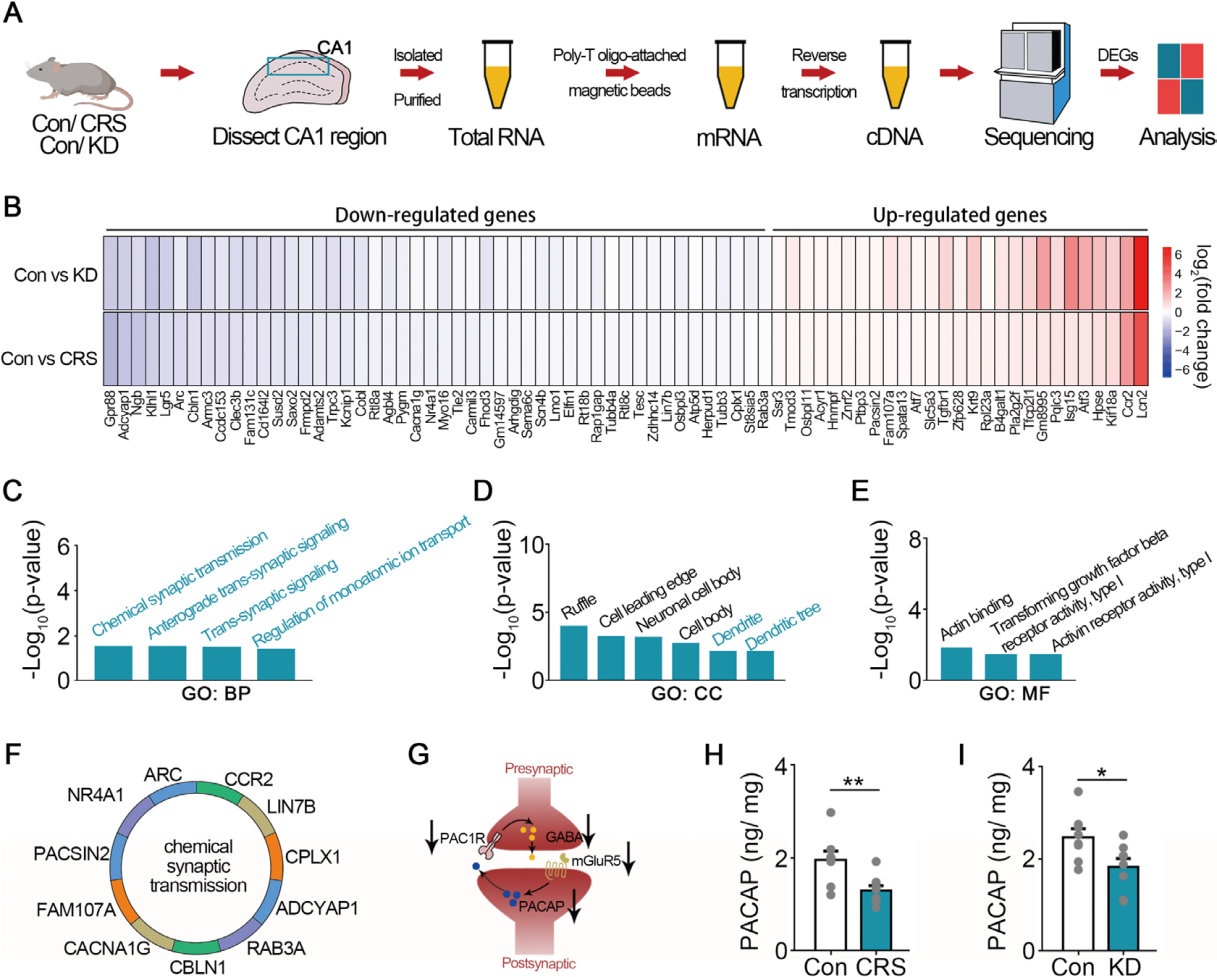
PACAP was predicted to be the downstream effector of chronic stress and mGluR5 knockdown‐induced memory deficits. (A) Schematic of RNA‐sequencing in hippocampal CA1 region. (B) The jointly down‐regulated and up‐regulated DEGs from RNA‐seq data after chronic stress or mGluR5 knockdown. (C) Gene Ontology biological process (BP) of the DEGs in (A). (D) Gene Ontology cellular components (CC) of the DEGs in (A). (E) Gene Ontology molecular function (MF) of the DEGs in (A). (F) The DEGs that were enriched in chemical synaptic transmission were listed. (G) Schematic diagram of the mGluR5‐PACAP‐GABAergic transmission pathway. (H) The PACAP level was reduced after chronic stress. *n* = 8 for each group. (I) The PACAP level was reduced after mGluR5 knockdown. *n* = 8 for each group. Error bars, mean ± SEM. *p < 0.05, **p < 0.01.

To understand the molecular mechanisms that contribute to impaired synaptic inputs and memory deficits after CRS or mGluR5 knockdown, we performed bulk RNA‐sequencing to achieve transcriptional alterations in the hippocampus after CRS or mGluR5 knockdown (Figure 4A, Figure S4A,B). In RNA‐Seq data, we first analyzed GABA‐related genes after chronic stress and mGluR5 knockdown. However, the relative expression of GABA synthase and GABA vesicular transporter exhibited no significant difference following chronic stress (Figure S5A–L). Next, we analyzed the jointly down‐regulated and up‐regulated differentially expressed genes (DEGs) from RNA‐seq data after chronic stress or mGluR5 knockdown, among which 48 DEGs were jointly down‐regulated and 27 DEGs were jointly up‐regulated (Figure 4B). GO enrichment analysis indicated that DEGs were significantly enriched in GO terms associated with chemical synaptic transmission (Figure 4C–E).

Among the DEGs that enriched in chemical synaptic transmission, pituitary adenylyl cyclase activating peptide (PACAP, gene symbol ADCYAP1) is a neuropeptide known to be important for modulating synaptic transmission, synaptic plasticity, and memory‐related behaviors (Figure 4F) [36, 37, 38, 39, 40, 41]. Therefore, PACAP might act as the downstream effector of chronic stress and mGluR5 knockdown‐induced memory deficits (Figure 4G). Moreover, PACAP could regulate GABAergic presynaptic vesicle release [42, 43]. Then, we measured the PACAP level in the hippocampal CA1 region after chronic stress or mGluR5 knockdown by ELISA. The results indicated that the PACAP level was reduced after chronic stress or mGluR5 knockdown (*chronic stress*: *t*(14) = 3.043, *p* = 0.0088, *Student's t‐test*; *mGluR5 knockdown*: *t*(14) = 2.476, *p* = 0.0267, *Student's t‐test*; Figure 4H,I).

### 3.5 PACAP Rescued Inhibitory Synaptic Input Deficits Following Chronic Stress

**FIGURE 5:**
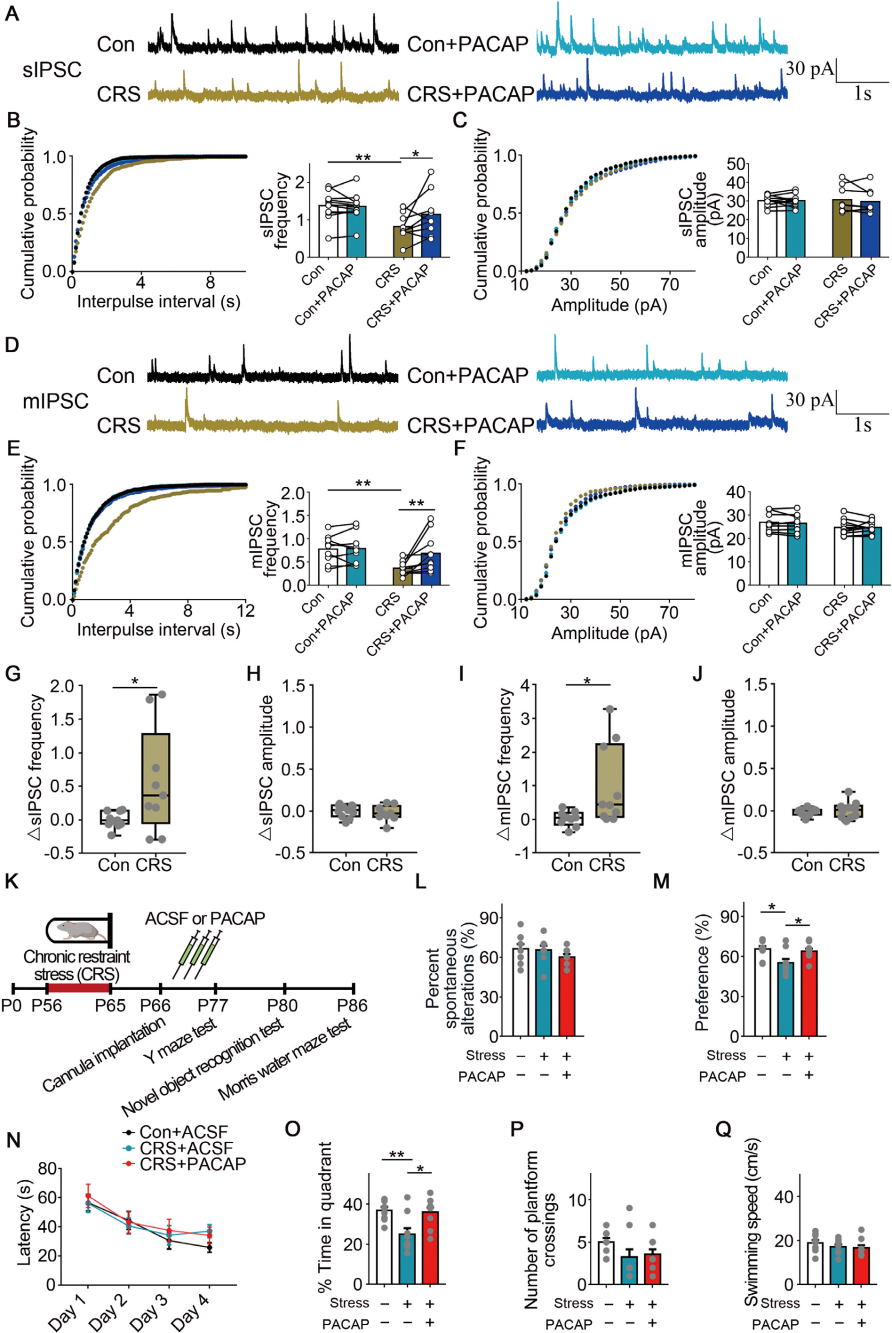
PACAP rescued GABAergic synaptic input deficits following chronic stress. (A–C) In sIPSC recordings, quantitative analysis of frequency and amplitude following PACAP application. 4 mice, *n* = 9–11 cells. (D–F) In mIPSC recordings, quantitative analysis of frequency and amplitude following PACAP application. 4 mice, *n* = 9–10 cells. (G, H) In sIPSC recordings, quantitative analysis of Δfrequency and Δamplitude following PACAP application. 4 mice, *n* = 9–11 cells. (I, J) In mIPSC recordings, quantitative analysis of Δfrequency and Δamplitude following PACAP application. 4 mice, *n* = 9–10 cells. (K) Diagram of behavioral assessments following chronic stress and PACAP infusion. (L) In the Y‐maze test, the mice infused with PACAP following chronic stress did not significantly differ from the control mice. There were 9, 9, 9 mice, separately. (M) In the NOR test, the mice infused with PACAP following chronic stress exhibited a restored preference for a new object. There were 9, 9, 9 mice, separately. (N–Q) In the MWM test, the mice infused with PACAP following chronic stress restored impaired memory. There were 9, 9, 9 mice, separately. Error bars, mean ± SEM. *p < 0.05, **p < 0.01.

PACAP could bind to its receptor PAC1R, after which it triggers the activation of downstream signaling cascades [53, 54, 55]. We investigated whether PACAP application might restore GABAergic synaptic input deficits, given that the level of PACAP was reduced following chronic stress. Following chronic stress, we recorded GABAergic synaptic inputs in CA1 pyramidal neurons of the hippocampus. Firstly, we tested if 5 nM PACAP application could affect GABAergic synaptic inputs in the control group. As reported, we also observed that 5 nM PACAP application increased GABAergic synaptic inputs (*t*(9) = 2.696, *p* = 0.0245, *Student's t‐test*; Figure S6A–C) [42, 43]. Our findings demonstrated that the frequency and amplitude of sIPSCs/mIPSCs in the control group were unaffected by 1 nM PACAP (Figure 5A–F). While there was no change in the amplitude, 1 nM PACAP raised the average values of frequency in the chronic stress group (*sIPSC*: *Con* vs. *CRS*: *p* = 0.0061. *CRS* vs. *CRS*(*PACAP*): *p* = 0.0181. *Con* vs. *CRS*(*PACAP*): *p* = 0.3171; *mIPSC*: *Con* vs. *CRS*: *p* = 0.0070. *CRS* vs. *CRS*(*PACAP*): *p* = 0.0031. *Con* vs. *CRS*(*PACAP*): *p* = 0.5882. *Repeated measure two‐way ANOVA, Fisher's LSD test for multiple comparisons corrections*; Figure 5A–F). Following 1 nM PACAP treatment, the curves of cumulative frequency distribution in sIPSC and mIPSC showed a rescue when examined by the KS test in the chronic stress group (Figure 5A–F). Additionally, after applying 1 nM PACAP, we found that the chronic stress group had a higher sIPSC frequency, although there was no change in the sIPSC amplitudes (*p* = 0.0381, *Mann–Whitney test*; Figure 5G,H). Similarly, following the PACAP application, we observed that mIPSC frequency was larger in the chronic stress group, while no change was observed in the mIPSC amplitude between the groups (*p* = 0.0133, *Mann–Whitney test*; Figure 5I,J). In summary, our data suggested that GABAergic synaptic input deficits could be restored by PACAP.

Furthermore, we locally infused PACAP into the dorsal hippocampal CA1 region in mice subjected to CRS and explored if this treatment could restore the memory deficits (Figure 5K). In the Y‐maze test, the spontaneous alternation percentage did not significantly differ across the groups (Figure 5L). In the novel object recognition test, the reduced preference for the novel object was rescued by PACAP application, indicating that recognition memory was rescued (*Con* vs. *CRS*: *p* = 0.0172, *CRS* vs. *CRS + PACAP*: *p* = 0.0475, *One‐way ANOVA test, Tukey test for multiple comparisons corrections*; Figure 5M). In the MWM test, the latency to search for the hidden platform showed no change between the groups during the training phase (Figure 5N). During the probe test, infusion of PACAP restored impaired memory after chronic stress (*F*_(2,24)_ = 7.41, *Con* vs. *CRS*: *p* = 0.0055, *CRS* vs. *CRS + PACAP*: *p* = 0.0107, *One‐way ANOVA, Tukey test*; Figure 5O). Meanwhile, no significant difference was found in the swimming velocity or crosses over the absent platform (Figure 5P,Q). Together, these data indicated that PACAP could restore memory deficits following chronic stress.

## 4 Discussion

**FIGURE 6:**
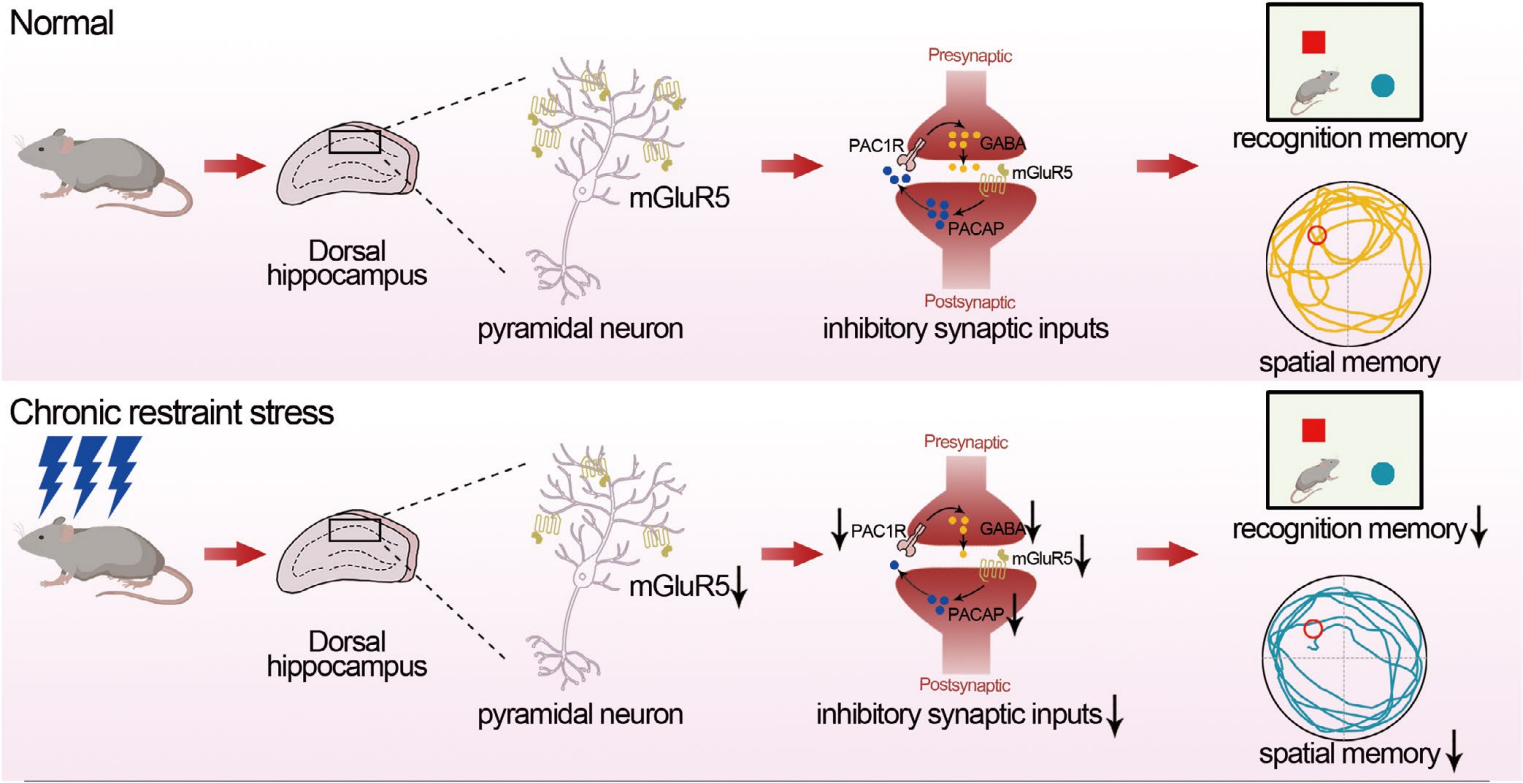
Diagrammatic representation of the role of mGluR5 in controlling memory impairments brought on by chronic stress. Chronic stress caused memory impairment, decreased GABAergic synaptic inputs, and decreased mGluR5 and PACAP levels in the hippocampus CA1. CDPPB restored memory damage by reversing the decline in GABAergic synaptic inputs caused by the stress. The reduction in GABAergic synaptic inputs and memory deficits brought on by chronic stress could also be reversed by PACAP administration.

In this study, our results showed that chronic restraint stress impaired hippocampal‐dependent memory. Chronic stress reduced mGluR5 level in the CA1 region, and mGluR5 knockdown also induced memory deficits. Meanwhile, GABAergic synaptic input deficits were induced by chronic stress or mGluR5 knockdown. The enhancement of mGluR5 activity rescued GABAergic synaptic input and memory deficits following chronic stress. Furthermore, after chronic stress and mGluR5 knockdown, we identified that PACAP was down‐regulated. GABAergic synaptic input deficits following chronic stress could be restored by PACAP application. In our work, these results suggested that the mGluR5 participates in regulating chronic stress‐induced memory disorders (Figure 6).

Chronic restraint stress is a commonly used mouse model to develop stress‐related diseases [56, 57, 58]. Previous studies show that stress could impair learning and memory in humans and mice [59, 60]. However, the involvement of mGluR5 in chronic stress‐induced memory deficits was unknown. Our results suggested that reduced mGluR5 level is responsible for chronic stress‐impaired memory, which was consistent with the previous findings that mGluR5 KO mice exhibited impaired learning and memory [61]. Following chronic stress, the CRS‐treated mice showed normal working memory in the Y‐maze test, but impaired object recognition memory in the NOR test and impaired spatial memory in the MWM test. There might be two reasons for the inconsistent performance across behavioral tests. First, the Y‐maze test reflects working memory, while the NOR test and MWM test reflect recognition memory and spatial memory. The effects of chronic stress might be different on different types of memory. Second, it took around 5 min to assess working memory (short‐term memory) in the Y‐maze test. However, it took 5 days to assess spatial memory (long‐term memory) in the MWM test and 3 days to assess object recognition memory in the NOR test. The effects of chronic stress might be different on short term and long term memory. The hippocampus is a typical brain area responsible for learning and memory. Evidence suggests that chronic stress could impair hippocampal function, including altered synaptic transmission, neuron excitability, neurogenesis, neuronal morphology, and cell death [62]. Consistently, our results also indicated that the hippocampus was a key brain area for stress‐impaired memory, which might be a target brain region for treating chronic stress‐impaired memory.

mGluR5 is distributed throughout the brain, including in the hippocampus. Previous studies suggest that mGluR5 in the hippocampus plays a key regulatory role in NMDAR‐dependent LTP, and mGluR5 KO mice show impaired learning and memory [61]. Also, mGluR5 is reported to be important for memory and LTP in the lateral amygdala [31]. In accordance with these studies, our results also suggested that hippocampal mGluR5 could regulate chronic restraint stress‐impaired memory. Together, these results suggested that mGluR5 may be a promising drug target to treat chronic stress‐impaired memory. There is a major limitation of our work: we did not explore the role of mGluR5 in female mice after chronic stress. Previous studies reveal that the contribution of mGluR5 is different between male and female when exposed to stress [63]. These results indicate that the stress‐induced memory deficits might not be due to mGluR5 in females. The role of mGluR5 in female mice needs further investigations.

Previous work (ours and others) showed that mGluR5 was declined and inhibitory synaptic transmission was impaired after chronic stress [21]. Therefore, we focused on mGluR5 and inhibitory synaptic transmission. It has also been reported that mGluR5 activation increases IPSC frequency, which shows that GABAergic synaptic inputs could be regulated by mGluR5 [64, 65]. Previous studies indicated that inhibition of mGluR5 could increase hippocampal BDNF level and postsynaptic BDNF could act as a retrograde synaptic regulator to reduce GABAergic input [66, 67, 68]. Similarly, in our study, we observed that PACAP level was reduced after chronic stress or mGluR5 knockdown. The reduced PACAP level might act as a retrograde synaptic regulator to reduce GABAergic input.

The excitatory synaptic inputs promote the flow of information in the hippocampus. Moreover, the excitatory synaptic inputs could induce synaptic plasticity, which is considered the cellular mechanism of memory formation [16]. During behavioral tests, LTP could be induced and improve the consolidation of memory. After CRS, our results indicated that excitatory synaptic inputs were reduced, which might affect the synaptic plasticity and information processing capability of pyramidal neurons. Then, the spatial memory in the MWM test was impaired after CRS. The hippocampus includes many types of GABAergic inhibitory interneurons, such as SOM (neuropeptide somatostatin), VIP (vasoactive intestinal polypeptide), and PV (calcium‐binding protein parvalbumin) positive cells, which could provide feedforward and feedback inhibition to pyramidal cells [16]. During spatial exploration, most CA1 interneurons are active [69]. Some studies indicated that the inactivation of PV positive cells led to working memory deficits [70]. Moreover, chemogenetic inhibition of the VIP positive cells led to impairments in both short‐term and long‐term memory in a spatial learning task [71]. Therefore, the inhibitory synaptic input is crucial for memory formation. How these inhibitory synaptic inputs work together to produce memory needs further investigation. After CRS, our results indicated that inhibitory synaptic inputs were reduced, which might affect the information processing capability of pyramidal neurons. Then, the spatial memory in the MWM test was impaired after CRS.

By transcriptome sequencing database, PACAP was filtered. We observed that PACAP levels were reduced after chronic stress or mGluR5 knockdown. Furthermore, GABAergic synaptic input deficits and memory deficits could be rescued by PACAP application following chronic stress. In our previous work [21], we demonstrated that only mGluR5 decrease could not lead to memory deficits. From our perspective, mGluR5‐mediated (not PACAP‐mediated) synaptic impairments could not lead to memory deficits. However, mGluR5‐PACAP‐mediated synaptic impairments might lead to memory deficits. As the molecular mechanisms for the synaptic impairments were different, the behavioral phenotypes might be different.

CDPPB is brain‐penetrant and could enhance the activity of mGluR5. It has been reported that, compared to vehicle‐treated controls, CDPPB significantly enhances memory and improves search strategy in the Barnes maze [49]. Meanwhile, CDPPB could enhance adaptive learning and recognition memory [51, 52]. Therefore, CDPPB might be a promising drug for treating chronic stress‐induced learning and memory disorders. CDPPB is a positive allosteric modulator for mGluR5. In our study, the mGluR5 level was reduced following chronic stress, and CDPPB enhanced the activity of mGluR5. Meanwhile, the reduced PACAP level following chronic stress might be rescued by enhanced mGluR5 activity. Then, the impaired inhibitory synaptic input following chronic stress might be restored by elevated PACAP level. Finally, the stress‐induced cognitive deficits were rescued by enhanced inhibitory synaptic input. At present, few studies focused on the interaction between CDPPB and PACAP. From our perspective, CDPPB could enhance the activity of mGluR5, and mGluR5 activation could increase PACAP level.

There is a limitation associated with our study. The restraint stress was applied daily in the resting phase for rodents. We could not rule out the potential confounding effects of sleep deprivation. In general, the sleep deprivation was performed for a continuous period of time (such as 24, 48, 72 h) [72]. Moreover, it has been reported that repeated periods of 4 h sleep deprivation during 5 days have no effect on avoidance learning, indicating that animals could adapt to the condition via sleep rebound over the rest of the day [72]. Therefore, from our perspective, the effects of restraint stress might not be due to the sleep deprivation.

## 5 Conclusions

In conclusion, we clarified that the mGluR5 is responsible for chronic restraint stress‐induced memory disorders. This pathway might provide potential drug targets for treating chronic stress‐induced learning and memory disorders.

## Author Contributions

X.L. proposed the concept and designed the experiments. H.‐C.L. and Z.‐J.D. performed the experiments. X.L., H.‐C.L., Z.‐J.D., and H.C. analyzed the results. X.L., H.‐C.L., T.G., and S.‐C.Y. interpreted the results. X.L. wrote and edited the manuscript.

## Ethics Statement

The animal protocols were approved by the Southern Medical University Animal Ethics Committee.

## Conflicts of Interest

The authors declare no conflicts of interest.

## Supporting information


Data S1.

Figures S1–S6.

## Data Availability

The data that support the findings of this study are available on request from the corresponding author.
