# Supplementary material for: mGluR5 in Pyramidal Neurons in the Hippocampus Mediates Chronic Stress‐Induced Memory Deficits
Source: CNS Neurosci Ther. 2025 Jun 12;31(6):e70477. doi: 10.1111/cns.70477 (PMC12163188; doi:10.1111/cns.70477)
Supplement: Supplementary file 1 — Data S1. [file CNS-31-e70477-s002.pdf]

A

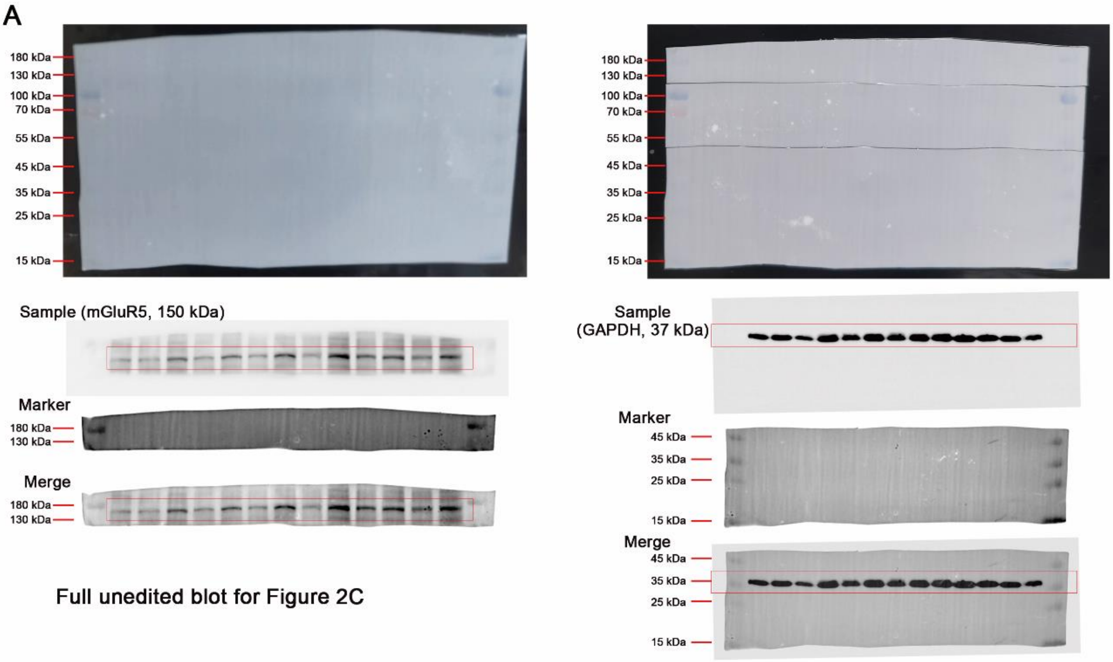

Full unedited blot for Figure 2C

B

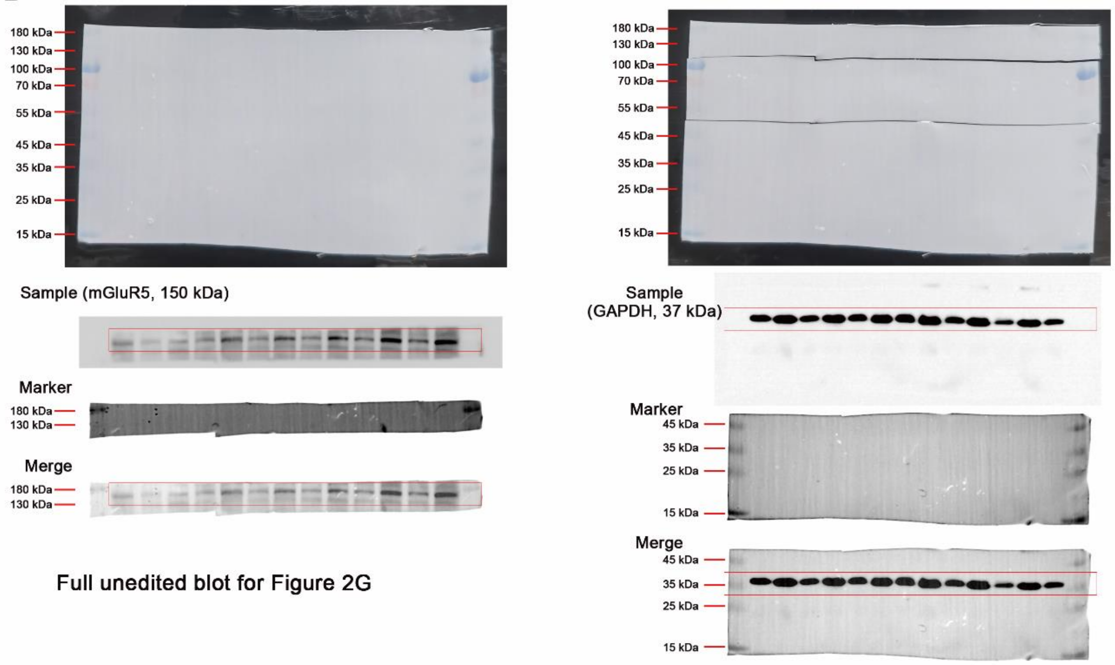

Full unedited blot for Figure 2G

C

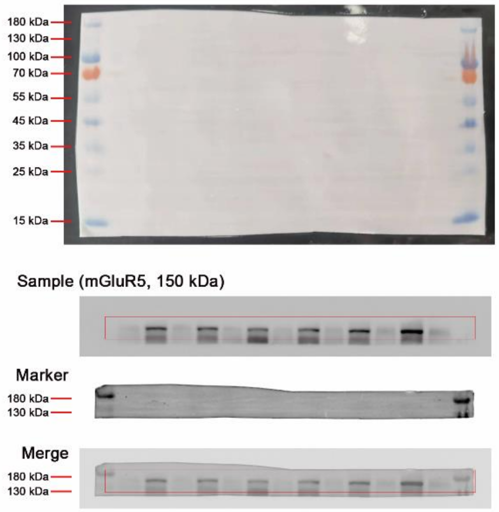

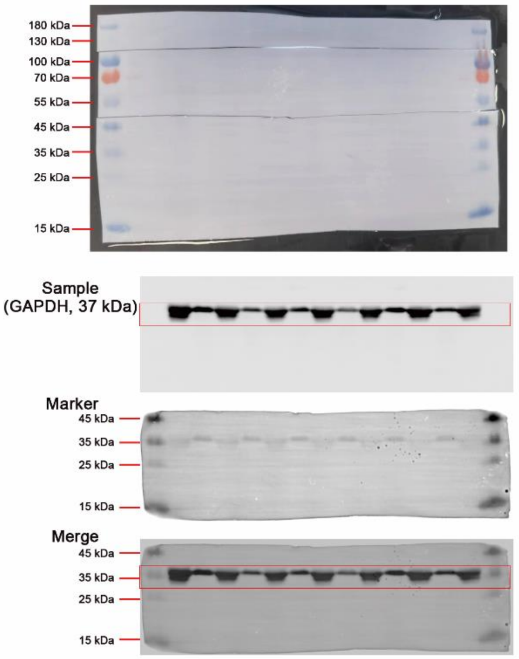

Full unedited blot for Figure S2A
