# Supplementary material for: mGluR5 in Pyramidal Neurons in the Hippocampus Mediates Chronic Stress‐Induced Memory Deficits
Source: CNS Neurosci Ther. 2025 Jun 12;31(6):e70477. doi: 10.1111/cns.70477 (PMC12163188; doi:10.1111/cns.70477)
Supplement: Supplementary file 2 — Figures S1–S6. [file CNS-31-e70477-s001.docx]

Supplementary Materials for

mGluR5 in pyramidal neurons in the hippocampus mediates chronic stress-induced memory deficits

Hong-Cheng Lu^3^, Zhuo-Jun Du^3^, Hao Chen^3^, Ting Guo^3^, Shu-Cai Yang^1,2^**,** Xin Li^1,2^

^1^Pingshan District Peoples' Hospital of Shenzhen, Shenzhen, Guangdong, P.R. China

^2^Pingshan Hospital, Southern Medical University, Shenzhen, Guangdong, P.R. China

^3^Key Laboratory of Mental Health of the Ministry of Education, Guangdong-Hong Kong-Macao Greater Bay Area Center for Brain Science and Brain-Inspired Intelligence, Guangdong-Hong Kong Joint Laboratory for Psychiatric Disorders, Guangdong Province Key Laboratory of Psychiatric Disorders, Guangdong Basic Research Center of Excellence for Integrated Traditional and Western Medicine for Qingzhi Diseases, Department of Neurobiology, School of Basic Medical Sciences, Southern Medical University, Guangzhou, China; State Key Laboratory of Organ Failure Research, Institute of Brain Diseases, Nanfang Hospital, Southern Medical University, China.

**Correspondence**

Xin Li, Pingshan District Peoples' Hospital of Shenzhen, Shenzhen, China. Pingshan Hospital, Southern Medical University, Shenzhen, China.

Email: m15626048599@163.com

**Supplementary text**

**
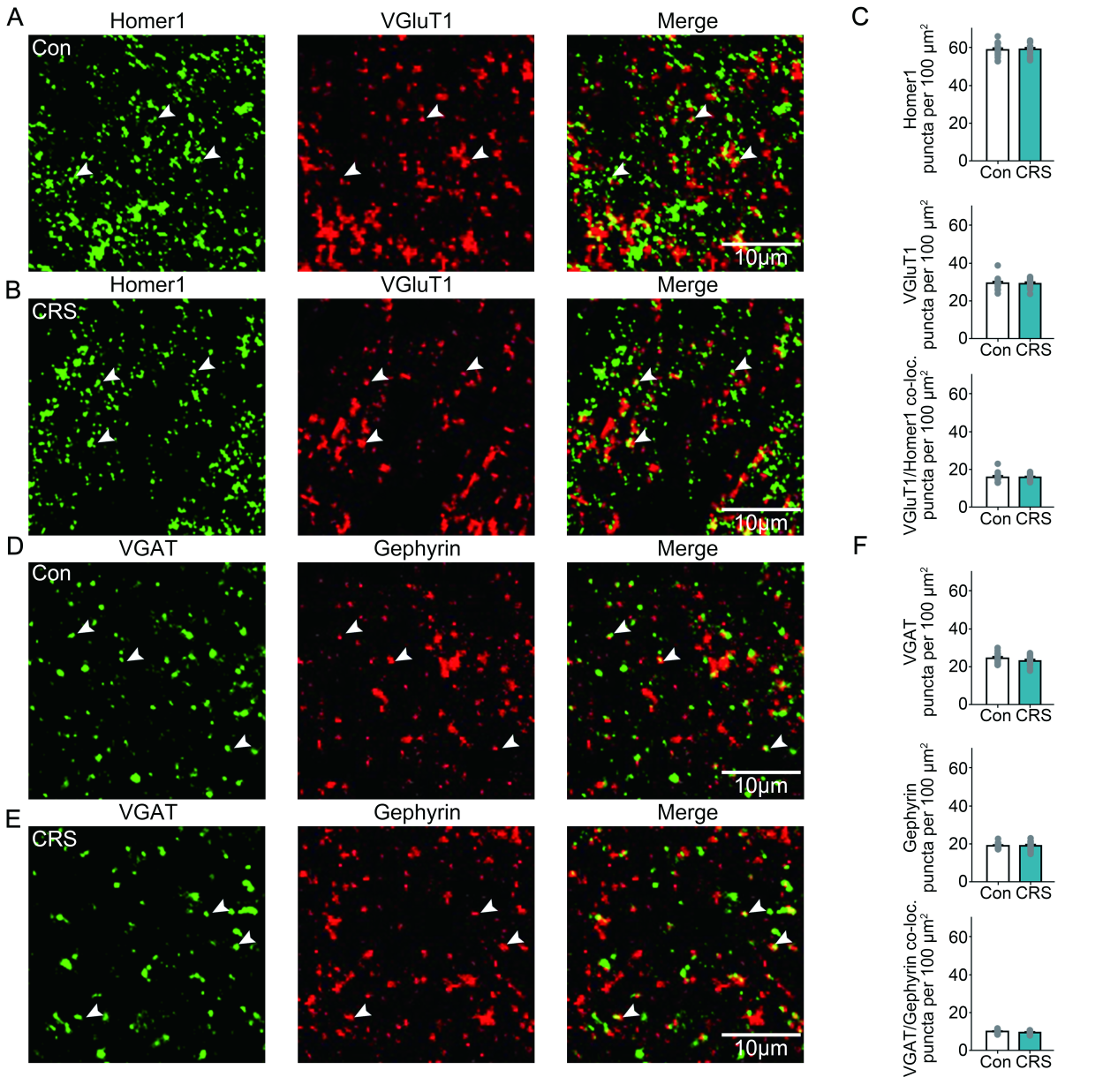
**

**Fig.S1. Chronic restraint stress did not decrease excitatory or inhibitory synaptic density in the hippocampal CA1 region. A-C** Representative images and quantitative analysis of the Homer1 and VGluT1 puncta in the hippocampal CA1 region. n=16 slices from 4 mice for each group. **D-F** Representative images and quantitative analysis of the VGAT and Gephyrin puncta in the hippocampal CA1 region. n=16 slices from 4 mice for each group. Error bars, mean ± SEM.


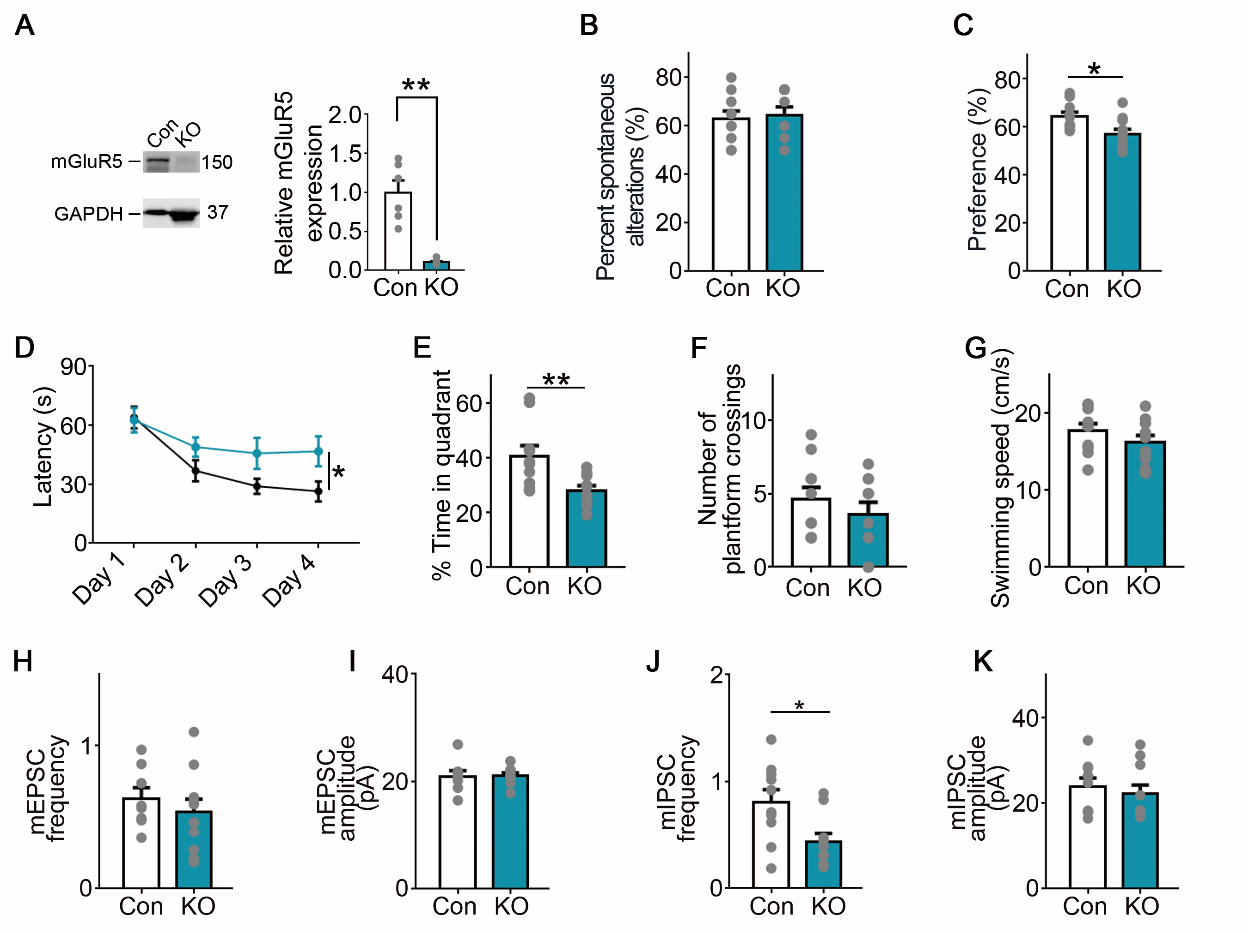


**Fig.S2. Behavioral tests of the mGluR5 KO mice. A** The specificity of the mGluR5 antibody was confirmed by western blot. There were 6-7 mice in each group. **B** The mGluR5 KO mice did not show a significant difference with control mice in the Y maze test. n=10 mice for each group. **C** The mGluR5 KO mice showed reduced preference for novel object in the novel object recognition test. n=10 mice for each group. **D** During the training phase in the Morris water maze, the mGluR5 KO mice exhibited increased latency to find the hidden platform when compared with control mice. n=10 mice for each group. **E** During the probe test, the mGluR5 KO mice showed impaired memory in the Morris water maze. **F, G** The mGluR5 KO mice showed no significant difference from the control mice in platform crossings and swimming speed. **H, I** In mEPSC recordings, quantitative analysis of frequency and amplitude after mGluR5 knockout. 4 mice, n=8-10 cells. **J, K** In mIPSC recordings, quantitative analysis of frequency and amplitude after mGluR5 knockout. 4 mice, n=10 cells for each group. Error bars, mean ± SEM.


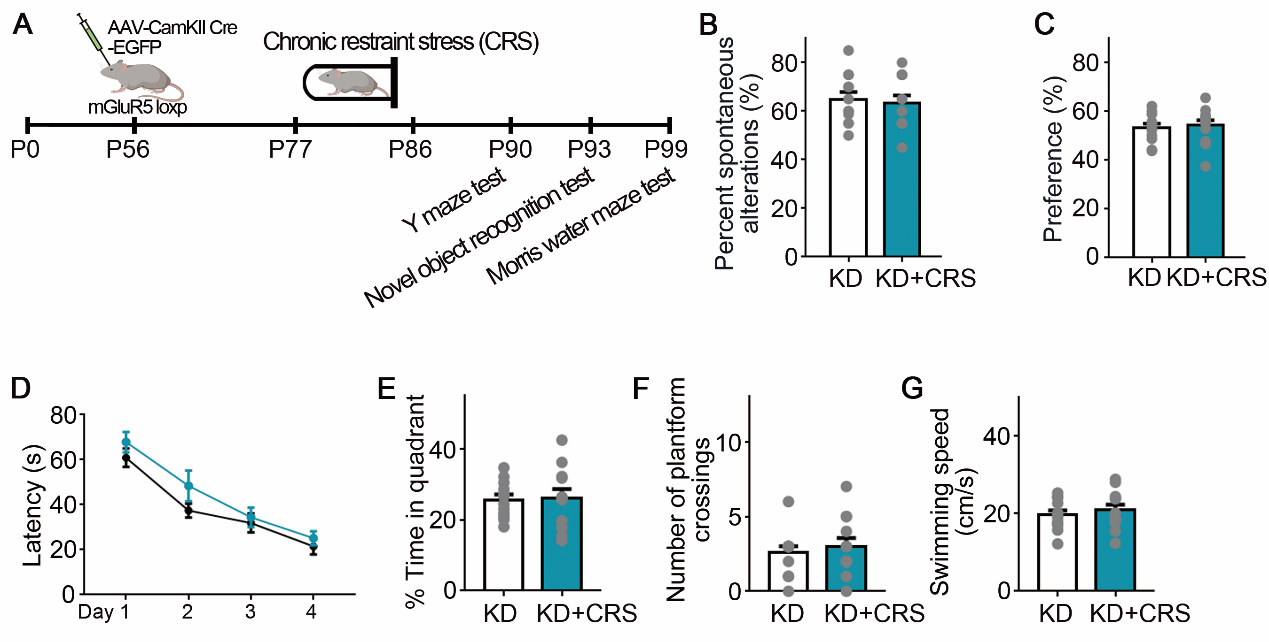


**Fig.S3. Memory deficits could not be further increased by chronic stress in mGluR5 knockdown mice. A** Diagram of behavioral assessments following virus injection and chronic stress. **B** In the Y maze test, the spatial working memory was not impaired after CRS in mGluR5 knockdown mice. n=12 mice for each group. **C** In the novel object recognition test, the recognition memory was not impaired after CRS in mGluR5 knockdown mice. n=12 mice for each group. **D** During the training phase in the Morris water maze, the latency to search for the hidden platform was unaltered after CRS in mGluR5 knockdown mice. n=12 mice for each group. **E-G** During the probe test, the spatial memory and swimming speed were unaltered after CRS in mGluR5 knockdown mice. n=12 mice for each group. Error bars, mean ± SEM.

**
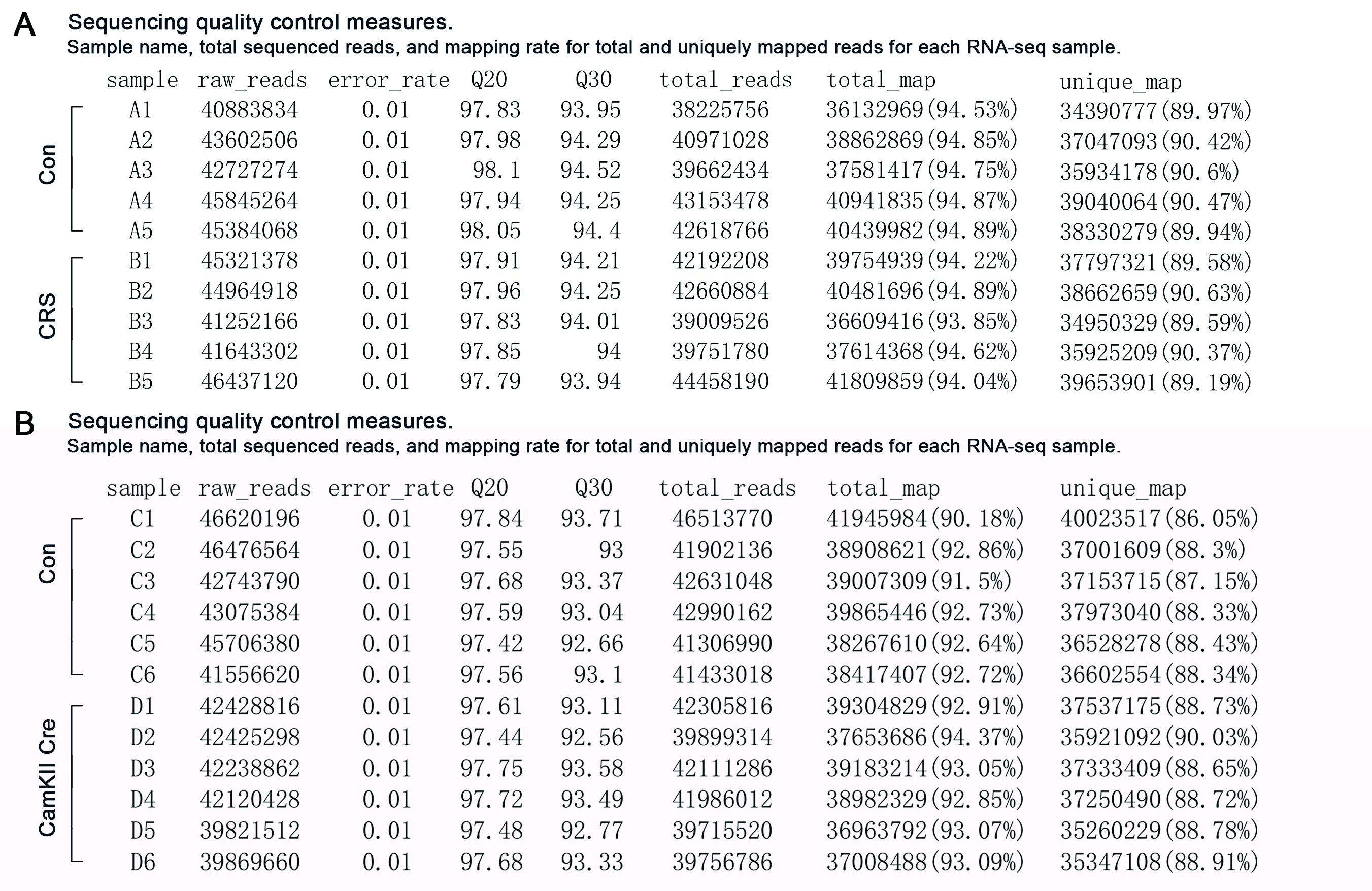
**

**Fig.S4. Sequencing quality control measures. A** Sample name, total sequenced reads, and mapping rate for total and uniquely mapped reads for each RNA-seq sample after chronic stress. **B** Sample name, total sequenced reads, and mapping rate for total and uniquely mapped reads for each RNA-seq sample after mGluR5 knockdown.


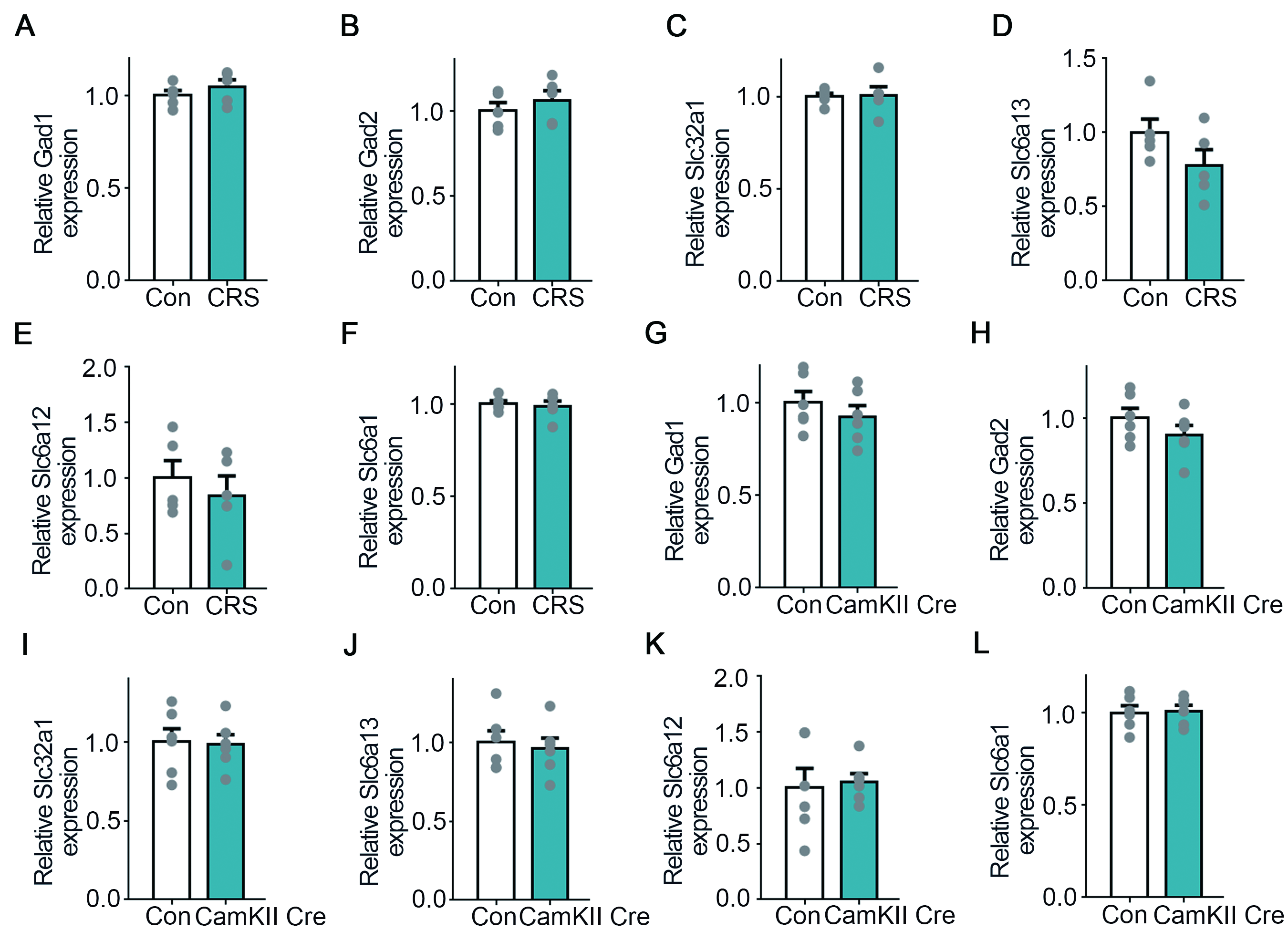


**Fig.S5. GABA-related genes were retrieved from RNA-seq data after chronic stress and mGluR5 knockdown. A-B** The mRNA level of GABA synthase after chronic stress. **C-F** The mRNA level of GABA vesicular transporter after chronic stress. **G-H** The mRNA level of GABA synthase after mGluR5 knockdown. **I-L** The mRNA level of GABA vesicular transporter after mGluR5 knockdown. Error bars, mean ± SEM.


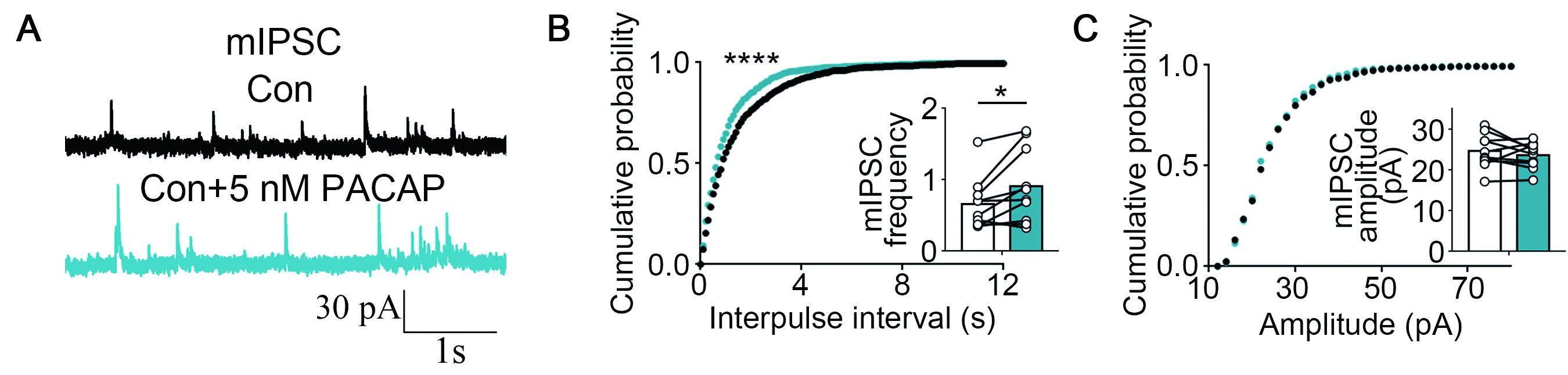


**Fig.S6. PACAP (5nM) increased inhibitory synaptic inputs in control group. A-C** Representative traces and quantitative analysis of mIPSCs after 5nM PACAP application. n=10 cells from 4 mice in the control group. No difference in mIPSC amplitude. Error bars, mean ± SEM.
